# High-fat/high-sucrose diet results in a high rate of MASH with HCC in a mouse model of human-like bile acid composition

**DOI:** 10.1097/HC9.0000000000000606

**Published:** 2024-12-11

**Authors:** Hajime Ueda, Akira Honda, Teruo Miyazaki, Yukio Morishita, Takeshi Hirayama, Junichi Iwamoto, Tadashi Ikegami

**Affiliations:** 1 Department of Internal Medicine, Division of Gastroenterology and Hepatology, Tokyo Medical University Ibaraki Medical Center, Ami, Ibaraki, Japan; 2 Joint Research Center, Tokyo Medical University Ibaraki Medical Center, Ami, Ibaraki, Japan; 3 Diagnostic Pathology Division, Tokyo Medical University Ibaraki Medical Center, Ami, Ibaraki, Japan

**Keywords:** chenodeoxycholic acid, Cyp2a12, Cyp2c70, FGF15, knockout mouse

## Abstract

**Background:**

Wild-type (WT) mice fed a conventional high-fat/high-sucrose diet (HFHSD) rarely develop metabolic dysfunction–associated steatohepatitis (MASH) with HCC. Because mouse bile acid (BA) is highly hydrophilic, we hypothesized that making it hydrophobic would lead to MASH with HCC.

**Methods:**

Eleven-week-old WT and *Cyp2a12/Cyp2c70* double knockout (DKO) mice were divided into two groups, including one which was fed a normal chow diet, and one which was fed an HFHSD. Samples were collected after 15, 30, 47, and 58 weeks for histological, biochemical, and immunological analyses.

**Results:**

In the HFHSD group, body weight gain did not differ in WT versus DKO mice, although HFHSD-fed DKO mice exhibited markedly accelerated liver inflammation, fibrosis, and carcinogenesis. HFHSD upregulated lipogenesis and downregulated fatty acid oxidation in both WT and DKO mice, which increased liver lipid accumulation and lipotoxicity. However, the increase in reactive oxygen species production and carcinogenesis observed in DKO mice could not be explained by abnormal lipid metabolism alone. Regarding BA metabolism, DKO mice had a higher hydrophobicity index. They exhibited an age-associated increase in chenodeoxycholic acid (CDCA) levels because of CYP8B1 activity inhibition due to the farnesoid X receptor activation. HFHSD further downregulated CYP8B1, presumably by activating the Liver X receptor. Liver CDCA accumulation was associated with increased inflammation, reactive oxygen species production, and hepatocyte FGF15 induction. Moreover, in noncancerous liver tissues, HFHSD appeared to activate STAT3, an oncogenic transcription factor, which was enhanced by a CDCA-rich environment.

**Conclusions:**

Here, we developed a new model of MASH with HCC using mice with human-like BA composition and found that HFHSD and elevated hepatic CDCA synergistically increased the risk of MASH with HCC.

## Graphical abstract

**Figure:**
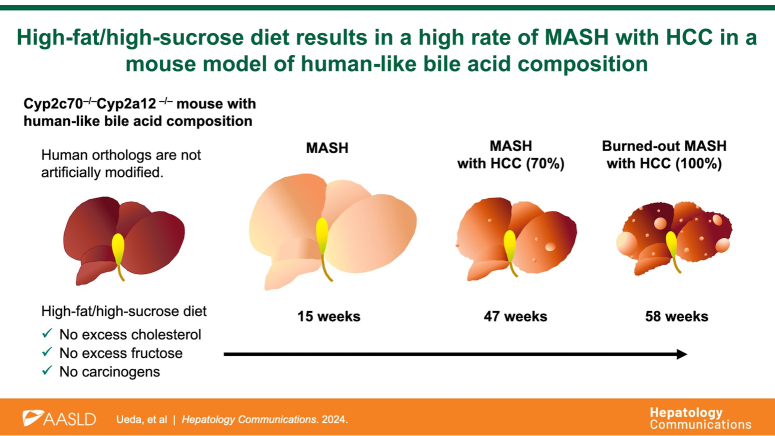

## INTRODUCTION

Although advances in antiviral therapy have led to declining rates of HCC, which is caused by viral hepatitis, the rates of HCC caused by metabolic dysfunction–associated steatotic liver disease (MASLD) have increased, making it a worldwide challenge. Globally, MASLD prevalence is estimated at 25%, with ~25% of its cases progressing to metabolic dysfunction–associated steatohepatitis (MASH). Furthermore, about 25% of MASH cases eventually progress to liver cirrhosis, with 2.6% of patients with MASH-related liver cirrhosis developing HCC annually.^1^,^2^ However, in humans, the factors underlying MASLD progression to MASH and HCC are unclear,^2^,^3^ and other than eliminating risk factors, strategies for preventing disease progression have not been established.

In addition to patient liver sample inaccessibility, the lack of animal models that fully recapitulate human MASLD has hindered progression in this field.^3^ The well-known MASH models, methionine-choline deficient^4^ and streptozotocin-treated type 1 diabetes mice,^5^ are lean, and they differ from human MASH etiologically and metabolically.^6^ Therefore, mouse models that, on a basic high-calorie diet, mimic the gradual MASLD to MASH to liver cirrhosis to HCC progression observed in humans are needed. When fed a high-fat diet or a high-fat/high-sucrose diet (HFHSD), without nonphysiological cholesterol or fructose loading, conventional mice rarely develop severe fibrosis or HCC.^6^,^7^ However, feeding a high-fat diet to major urinary protein-urokinase-type plasminogen activator transgenic mice, which have hepatocyte endoplasmic reticulum stress because of high urokinase-type plasminogen activator expression, induces MASH with HCC,^8^ indicating that in mice fed a basic high-calorie diet, an additional factor is required to promote MASH and HCC development.

Bile acids (BAs), cholesterol catabolism end products, are essential for intestinal digestion and absorption, and they are recycled through enterohepatic circulation. BA composition is markedly different in humans versus mice, and in mice, it is more hydrophilic and cytoprotective than in humans. While mouse CYP2C70 metabolizes the hydrophobic primary BA, chenodeoxycholic acid (CDCA), into the very hydrophilic, muricholic acids, CYP2A12 reverts hydrophobic secondary BAs into less hydrophobic primary BAs. However, humans lack these enzymes. Therefore, as in humans, in mice fed a basic high-calorie diet, hydrophobic and cytotoxic BA composition may promote MASH and HCC.

To test this hypothesis, we generated *Cyp2a12*^*–/–*^ *Cyp2c70*^*–/–*^ double knockout (DKO) mice, which have a human-like hydrophobic BA composition,^9^ and treated them with basic HFHSD without cholesterol or fructose loading. Although these mice exhibited less severe hepatic steatosis and insulin resistance when compared with wild-type (WT) mice, they had markedly accelerated liver inflammation, fibrosis, and carcinogenesis. Our findings suggest strongly that along with HFHSD, hepatic hydrophobic BA accumulation, especially CDCA, promotes MASH/HCC development.

## METHODS

### Animals

C57BL/6J WT and *Cyp2a12*^*–/–*^ *Cyp2c70*^*–/–*^ DKO mice (C57BL/6J background) were developed as described.^9^ Ursodeoxycholic acid (18.75 mg/100 mL) was administered through drinking water (from weaning to 9 weeks of age) to prevent liver injury in DKO mice.^10^ Male, 11-week-old, WT mice (n = 43) and DKO mice (n = 60) were divided into the normal diet and the HFHSD groups. Mice were maintained under regular 12-hour light/dark cycles (light period: 6:00 am–6:00 pm) in pathogen-free conditions. The nutritional contents of both diets are shown in Supplemental Table S1, http://links.lww.com/HC9/B797. After 15, 30, 47, and 58 weeks, mice were fasted overnight (with free access to water) and then euthanized through exsanguination under anesthesia with medetomidine, midazolam, and butorphanol during the middle of the light period (between 11:00 am and 3:00 pM). Next, serum, gallbladder, liver, small intestine, and fecal samples were collected and stored at −80°C. Ethical approval for the study was granted by the Animal Experiment Committee of Tokyo Medical University (approval numbers R2-0013, R3-0010, R4-037, and R5-013).

### Liver function tests

The serum levels of ALT, AST, and ALP were determined through colorimetric assays using Transaminase CII-Test and LabAssay ALP kits (FUJIFILM Wako Pure Chemical).

### Insulin resistance analysis

After overnight fasting, serum glucose and insulin levels were quantified using a Glucose CII-Test kit (FUJIFILM Wako) and an ELISA kit (Mercodia), respectively. Homeostatic model assessment for insulin resistance (HOMA-IR) was determined using the following equation: HOMA-IR = (fasting glucose [mmol/L] × fasting insulin [IU/L])/22.51. The glucose tolerance test and insulin tolerance test were performed by intraperitoneally injecting mice with glucose (2 g/kg of body weight) and regular insulin (0.75 U/kg of body weight), respectively,^11^ followed by tail vein blood glucose measurement using a Medisafe FIT glucometer (Terumo).

### Lipid analyses

The levels of total cholesterol, free cholesterol, triglycerides (TG), and free fatty acids (FFA) in sera and liver samples were measured using Cholesterol E-Test, Free Cholesterol E-Test, Triglyceride E-Test, and NEFA C-Test kits (All from FUJIFILM Wako), respectively. Serum lipopolysaccharide (LPS) levels were quantified using a ToxinSensor Chromogenic LAL Endotoxin Assay Kit (GenScript). Liver oxysterol levels were determined as described.^9^ Serum lipoprotein profiles were determined using gel permeation HPLC by Skylight Biotech Inc.^12^

### BA analysis

Liver, bile, small intestine, fecal, and serum BAs were analyzed using HPLC-tandem mass spectrometry as described.^9^

### Oxidative stress assays

The levels of liver 8-hydroxydeoxyguanosine (8-OHdG) and 4-hydroxynonenal (4-HNE), as well as mitochondrial and microsomal reactive oxygen species (ROS), were determined as described in the Supplemental Materials and Methods, http://links.lww.com/HC9/B797.

### Organic compound quantification

The serum levels of 3-hydroxybutyrate, 3-hydroxyisobutyrate, carnitine, acetylcarnitine, choline, trimethylamine *N*-oxide, and taurine, as well as liver palmitoylcarnitine levels, were quantified using HPLC-tandem mass spectrometry (Supplemental Materials and Methods, http://links.lww.com/HC9/B797).

### Histopathology

For formalin-fixed paraffin-embedded sections, hematoxylin and eosin, Masson’s trichrome, and immunohistochemical staining were used for liver histopathological examination (Supplemental Materials and Methods, http://links.lww.com/HC9/B797), while oil Red O staining was used for frozen sections.

### Gene expression analysis

Total RNA extraction, reverse transcription, and real-time quantitative PCR were done as described,^9^ and the primer sequences are shown in Supplemental Table S2, http://links.lww.com/HC9/B797. RNA-seq analysis was done as described in Supplemental Materials and Methods, http://links.lww.com/HC9/B797.

### Enzyme assays

The activities of CYP7A1 and CYP8B1 were measured in liver microsomes as described.^9^

### ELISA

Serum FGF15, FGF21, glucagon-like peptide-1 (GLP-1), and adiponectin levels, as well as liver TNFα, IL-1β, IL-6, TGFβ, IFN-γ, phospho-Tyr705, and total STAT3, and phospho-Ser536 and total NF-κB concentrations, were quantified using mouse ELISA kits as per manufacturer guidelines. The kit catalog numbers and supplier information are provided in Supplemental Materials and Methods, http://links.lww.com/HC9/B797.

### Statistical analyses

Data are presented as mean ± SEM. Statistical differences between groups were compared using 1-way ANOVA with a post hoc Tukey-Kramer test or Kruskal-Wallis with a post hoc Dunn-Bonferroni test. *p* < 0.05 was considered statistically significant. All statistical analyses were conducted on GraphPad Prism version 10.2.3 (GraphPad).

## RESULTS

### Insulin resistance is milder in DKO mice than in WT mice

**FIGURE 1:**
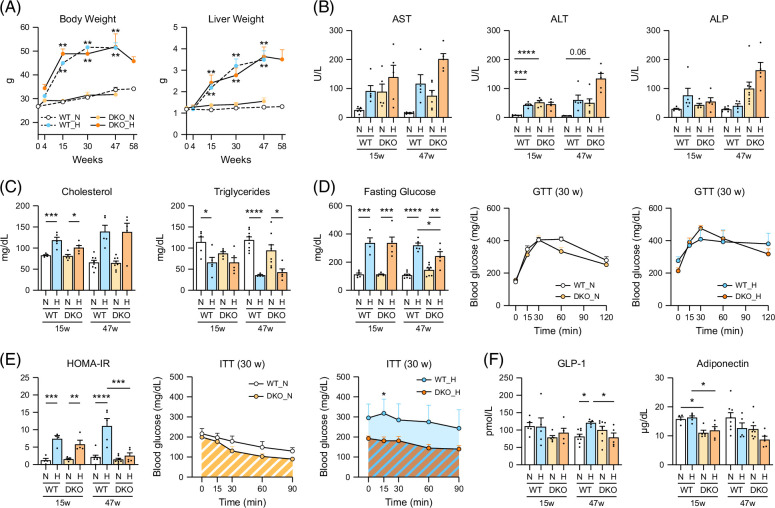
Physiological and metabolic effects of HFHSD in male WT and *Cyp2a12/Cyp2c70* DKO mice over 58 weeks. Starting at 11 weeks of age, mice were fed a normal diet or the HFHSD, and analyses were made after 15, 30, and 47 weeks. Sample sizes: n = 5/group at weeks 15 and 30, n = 5–8/group at week 47. (A) Body and liver weight changes over time. (B) Serum AST, ALT, and ALP activities. (C) Serum cholesterol and triglyceride concentrations. (D) Serum glucose levels and GTT results. (E) HOMA-IR and ITT results. (F) Serum GLP-1 and adiponectin levels. Data are presented as mean ± SEM. *, **, ***, and **** indicate *p* < 0.05, <0.01, <0.001, and <0.0001, respectively, based on 1-way ANOVA with a post hoc Tukey-Kramer test. Abbreviations: DKO, double knockout; GLP-1, glucagon-like peptide-1; GTT, glucose tolerance test; H, HFHSD; HFHSD, high-fat/high-sucrose diet; HOMA-IR, homeostatic model assessment for insulin resistance; ITT, insulin tolerance test; N, normal diet; WT, wild-type.

HFHSD-associated body and liver weight increase did not differ significantly between WT and DKO mice (Figure 1A). HFHSD was associated with elevated blood liver enzyme levels, which was more severe in DKO mice (Figure 1B), as well as with significantly elevated total cholesterol levels (Figure 1C). Normal mouse LDL-cholesterol levels account for 10%–15% of HDL-cholesterol, while in HFHSD mice, it was elevated to nearly 50% (Supplemental Figure S1A, http://links.lww.com/HC9/B797). However, in both genotypes, HFHSD was associated with decreased TG and FFA levels (Figure 1C and Supplemental Figure S1B, http://links.lww.com/HC9/B797).

Glucose intolerance analysis based on fasting serum glucose and glucose tolerance test assays (Figure 1D) revealed that HFHSD was associated with glucose intolerance in WT and DKO mice, which did not differ significantly between the 2 genotypes. Insulin resistance analysis based on HOMA-IR and insulin tolerance test assays (Figure 1E) revealed that HFHSD-caused insulin resistance was milder in DKO mice than in WT mice. Although insulin secretion or sensitivity is increased by the bioactive compounds, GLP-1, adiponectin, and FGF21, their fasting levels were not increased in DKO versus WT mice (Figure 1F and Supplemental Figure S1C, http://links.lww.com/HC9/B797).

### HFHSD-fed DKO mice exhibit accelerated liver fibrosis because of steatohepatitis

**FIGURE 2:**
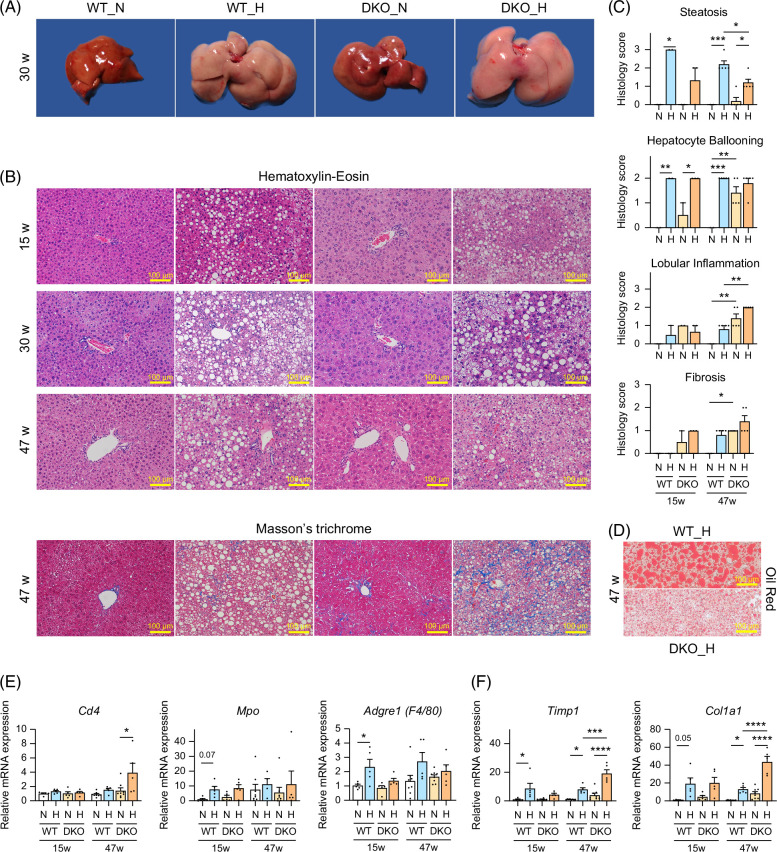
Liver appearance, pathology, and inflammation and fibrosis progression in male WT and *Cyp2a12/Cyp2c70* DKO mice fed the indicated diets. (A) Gross images of livers from WT and DKO mice fed an ND or an HFHSD for 30 weeks. (B) Representative microscopy images of livers from WT and DKO mice fed an ND and an HFHSD for 15, 30, and 47 weeks. (C) Histological steatosis, hepatocyte ballooning, lobular inflammation, and fibrosis scoring across various time points and diets. (D) Representative images of liver sections stained with Oil Red O to visualize lipid accumulation. (E,F) Liver mRNA expression levels of inflammation and fibrosis markers. Data are presented as mean ± SEM. *, **, ***, and **** indicate *p* < 0.05, <0.01, <0.001, and <0.0001, respectively, based on 1-way ANOVA with a post hoc Tukey-Kramer test. Abbreviations: *Adgre1*, adhesion G protein–coupled receptor E1 (formerly known as F4/80); *Cd4*, cluster of differentiation 4; *Col1a1*, collagen type I alpha 1 chain; DKO, double knockout; H, HFHSD; HFHSD, high-fat/high-sucrose diet; *MPO*, myeloperoxidase; N, ND; ND, normal diet; WT, wild-type.

HFHSD administration was associated with the livers of WT and DKO mice becoming pale orange (Figure 2A). Microscopic examination revealed that in both genotypes, 15 weeks of HFHSD led to steatosis and hepatocellular ballooning, with Mallory-Denk bodies, although lobular inflammation was mild (Figures 2B, C and Supplemental Figure S2A, http://links.lww.com/HC9/B797). However, after HFHSD for 47 weeks, DKO mice exhibited typical MASH findings, with worsening lobular inflammation. The gene expression levels of *Cd4*, myeloperoxidase (*Mpo*), and adhesion G protein–coupled receptor E1 (*Adgre1*, F4/80), which are helper T cell, neutrophil, and macrophage markers, support age-dependent inflammation progression, especially in DKO mice (Figure 2E).

Interestingly, when compared with WT mice, steatosis was milder in DKO mice. Although both genotypes developed macrovesicular steatosis by week 30 after HFHSD feeding, in DKO mice, it changed to microvesicular steatosis after 47 weeks (Figure 2D). Finally, in DKO mice, lipid droplets disappeared after 58 weeks, resulting in burned-out MASH (Supplemental Figure S2B, http://links.lww.com/HC9/B797). In addition, Masson’s trichrome staining, as well as *Timp1* and collagen type Iα1 (*Col1a1*) gene expression analyses, indicated that fibrosis progression was likelier in DKO mice than in WT mice (Figures 2B, F).

### CDCA accumulates in the livers of HFHSD-fed DKO mice

**FIGURE 3:**
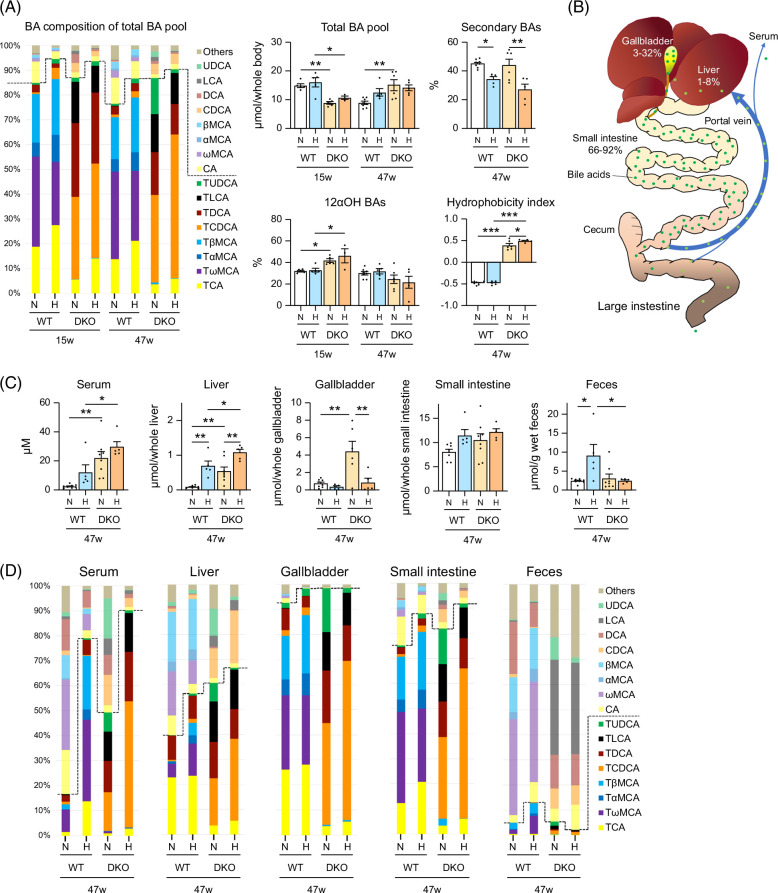
BA pool dynamics and composition in male WT and *Cyp2a12/Cyp2c70* DKO mice fed the indicated diets. (A) BA composition and total enterohepatic circulation BA pool levels, 12α-hydroxy (12α-OH) BA and secondary BA percentages, and hydrophobicity indices after 15 and 47 weeks. (B) Enterohepatic circulation BA distribution. (C, D) BA levels and compositions in serum, liver, gallbladder, small intestine, and feces after 47 weeks. Data are presented as mean ± SEM. *, **, and *** indicate *p* < 0.05, <0.01, and <0.0001, respectively, based on 1-way ANOVA with a post hoc Tukey-Kramer test. Abbreviations: BA, bile acid; DKO, double knockout; H, high-fat/high-sucrose diet; N, normal diet; WT, wild-type.

To determine the size and composition of the enterohepatic circulation BA pool, we summed the levels of each BA in the liver, gallbladder, and small intestines (Figure 3A and Supplemental Table S3, http://links.lww.com/HC9/B797). At 15 weeks, when compared with DKO mice, WT mice had larger total pool sizes than DKO mice, which decreased with age in WT mice, while increasing in DKO. Indeed, at 47 weeks, the sizes were larger in DKO mice than in WT mice. Although HFHSD tended to increase pool sizes, the effects were not statistically significant.

Regarding BA composition, at 15 weeks, the percentages of 12α-hydroxylated BAs, cholic acid and deoxycholic acid, were higher in DKO mice than in WT mice, and they exhibited an age-dependent decrease in DKO mice only. In addition, in both types of mice, HFHSD significantly reduced the percentages of secondary BAs. Consequently, in DKO mice fed with HFHSD for 47 weeks, CDCA levels increased to more than half of the BA pool (Figure 3A and Supplemental Table S3, http://links.lww.com/HC9/B797). When compared with WT mice, BA hydrophobicity indices were much higher in DKO mice, although HFHSD did not necessarily yield constant changes.

Enterohepatic circulation BAs were highest in the small intestine and lowest in the liver (Figure 3B and Supplemental Table S4, http://links.lww.com/HC9/B797). However, hepatic BA levels tended to increase in HFHSD-fed DKO mice (Figure 3C and Supplemental Figure S3B, http://links.lww.com/HC9/B797). Regarding BA composition, HFHSD increased the proportion of serum and liver conjugated-BAs, with taurine-conjugated CDCA (TCDCA) being the most abundant BA in the livers of DKO mice fed HFHSD for 47 weeks.

### CYP8B1 is markedly downregulated in HFHSD-fed DKO mice, probably through farnesoid X receptor and LXR coactivation

**FIGURE 4:**
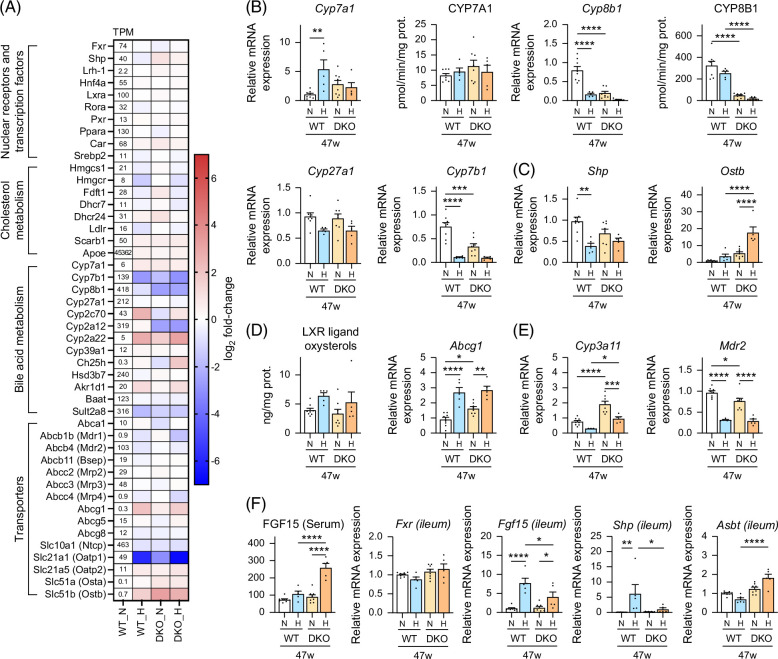
Liver cholesterol and BA metabolism regulation in male WT and *Cyp2a12/Cyp2c70* DKO mice fed the indicated diets. (A) RNA sequencing was used to determine the relative mRNA levels of genes involved in cholesterol and BA metabolism in WT and DKO mice fed an ND or an HFHSD for 30 weeks (n = 3/group). The heatmap presents log 2-fold changes when compared with ND-treated WT mice. (B) Liver mRNA expression levels and activities of key enzymes in the BA biosynthesis pathways after 47 weeks. (C) Liver mRNA levels of FXR target genes after 47 weeks. (D) Hepatic LXR ligand oxysterol levels and the mRNA levels of the LXR target gene, *Abcg1,* after 47 weeks. (E) Liver mRNA levels of the PXR target gene, *Cyp3a11*, and the PPARα target gene, *Mdr2,* after 47 weeks. (F) Serum FGF15 levels and ileal mRNA levels of *Fxr* and FXR target genes after 47 weeks. Data are presented as mean ± SEM. *, **, ***, and **** indicate *p* < 0.05, <0.01, <0.001, and <0.0001, respectively, based on 1-way ANOVA with a post hoc Tukey-Kramer test. Abbreviations: *Abcg1*, ATP-binding cassette transporter G1; *Asbt*, apical sodium-dependent bile acid transporter; BA, bile acid; DKO, double knockout; FXR, farnesoid X receptor; H, HFHSD; HFHSD, high-fat/high-sucrose diet; ​​​​​​LXR, liver X receptor; ​​​​​*Mdr2*, multidrug resistance protein 2; N, ND; ND, normal diet; ​​​​​​*Ostb*, organic solute transporter β; PPARα, peroxisome proliferator–activated receptor α; PXR, pregnane X receptor; ​​​​​​*Shp*, small heterodimer partner; TPM, transcripts per million; WT, wild-type.

RNA-seq analysis of relative cholesterol and BA metabolism gene expression in mouse livers treated for 30 weeks (Figure 4A) revealed that HFHSD and genetic modification (DKO) affected several genes, such as *Cyp8b1*, *Cyp7b1*, *Oatp1*, and *Ostb* similarly. Further analysis, with a focus on several essential genes, revealed that in both genotypes, HFHSD for 15 weeks markedly induced *Cyp7a1*, the rate-limiting enzyme in the classic BA biosynthesis pathway, although its expression was not significantly different in WT versus DKO mice (Figure 4B and Supplemental Figure S4A, http://links.lww.com/HC9/B797). However, genotypes or diet did not significantly affect the enzymatic activity of CYP7A1. In contrast, genetic modification (DKO) and HFHSD suppressed the gene expression and enzymatic activity of CYP8B1, the rate-limiting enzyme in cholic acid biosynthesis. *Cyp27a1* and *Cyp7b1* are key enzymes in the alternative BA biosynthetic pathway. Although no differences were observed in *Cyp27a1* expression, like *Cyp8b1*, *Cyp7b1* expression was suppressed in DKO mice, while HFHSD inhibited its enzymatic activity in both genotypes.

**FIGURE 5:**
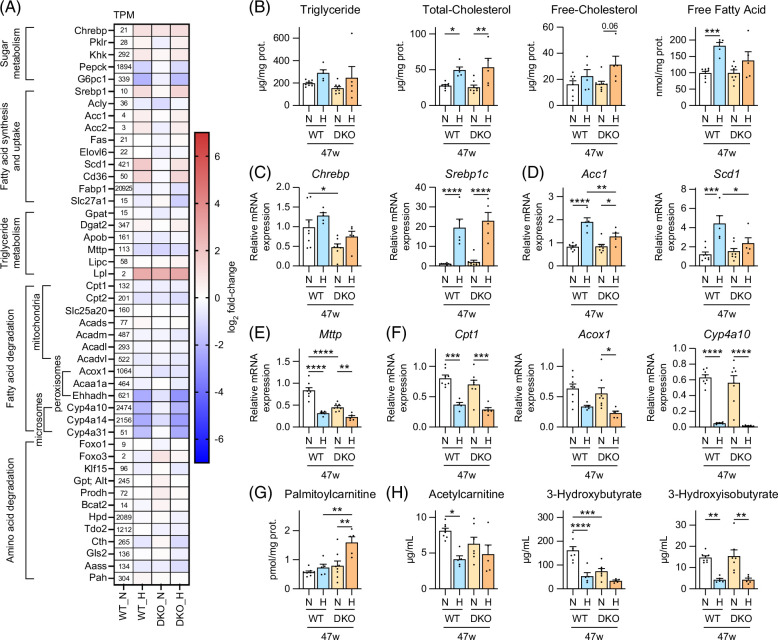
Liver sugar, lipid, and amino acid metabolism regulation in male WT and *Cyp2a12/Cyp2c70* DKO mice fed the indicated diets. (A) RNA sequencing was used to determine the relative mRNA levels of genes involved in sugar, fatty acid, and amino acid metabolism in WT and DKO mice fed an ND or an HFHSD for 30 weeks (n = 3/group). The heatmap presents log 2-fold changes when compared with ND-fed WT mice. (B) Liver lipid concentrations after 47 weeks expressed as per mg of protein. (C) Liver mRNA levels of fatty acid biosynthesis transcription factors, (D) key enzymes in de novo fatty acid synthesis, (E) *Mttp*, which is crucial for VLDL synthesis, and (F) essential enzymes in fatty acid β- and ω-oxidation after 47 weeks. (G) Liver levels of palmitoylcarnitine, a key intermediate in mitochondrial β-oxidation after 47 weeks. (H) The serum levels of markers involved in fatty acid β-oxidation and amino acid degradation after 47 weeks. Data are presented as mean ± SEM. *, **, ***, and **** indicate *p* < 0.05, <0.01, <0.001, and <0.0001, respectively, based on 1-way ANOVA with a post hoc Tukey-Kramer test. Abbreviations: *Acc1*, acetyl-CoA carboxylase 1; *Acox1*, acyl-CoA oxidase 1; *Chrebp*, carbohydrate-responsive element-binding protein; *Cpt1*, carnitine palmitoyltransferase 1; DKO, double knockout; H, HFHSD; HFHSD, high-fat/high-sucrose diet; *Mttp*, microsomal triglyceride transfer protein; ​​​​N, ND; ND, normal diet; *Scd1*, stearoyl-CoA desaturase 1; *Srebp1c*, sterol regulatory element-binding protein 1c; TPM, transcripts per million; WT, wild-type.

To assess the activation status of the farnesoid X receptor (FXR), we examined the expression of *Shp* and *Ostb*, which are direct FXR target genes (Figure 4C and Supplemental Figure S4B, http://links.lww.com/HC9/B797). However, it is difficult to explain FXR activity based on *Shp* expression changes since it is regulated by several factors, including inflammation, FFA, and pregnane X receptor.^9^,^13^ In contrast, *Ostb* expression reflected liver BA levels and their hydrophobicity well. In mice, liver X receptor α (LXRα) activation upregulates *Cyp7a1* directly and probably downregulates *Cyp7b1* and *Cyp8b1* by antagonizing retinoid-related orphan receptor α activity, probably due to competition for coactivators.^14^,^15^ The levels of the hepatic LXR ligand, oxysterol, and the expressions of its target genes, *Abcg1* and sterol regulatory element-binding protein 1c (Srebp1c), indicate HFHSD-mediated LXRα activation in both genotypes (Figures 4D and 5C, Supplemental Figures S4C and S5C, http://links.lww.com/HC9/B797, and Supplemental Table S5, http://links.lww.com/HC9/B797). It should be mentioned here that SREBP1c upregulates Cyp8b1.^16^ However, the inhibitory effects of FXR and LXR likely exceeded the upregulation by Srebp1c in HFHSD-fed mice. To evaluate pregnane X receptor and peroxisome proliferator–activated receptor α (PPARα) activation, we evaluated *Cyp3a11* and *Mdr2* expression (Figure 4E and Supplemental Figure S4D, http://links.lww.com/HC9/B797). Because lithocholic acid is a potent pregnane X receptor ligand, when compared with WT mice, DKO mice had higher *Cyp3a11* expression. In addition, in both genotypes, HFHSD inhibited *Cyp3a11*, which is suppressed by LXR activation.^17^ Moreover, HFHSD downregulated *Mdr2* expression, suggesting that LXR activation inhibits PPARα activity.

We evaluated intestinal FXR activation by measuring serum FGF15 and FXR-related gene mRNA levels in the distal ileum (Figure 4F). Ileal *Fgf15* and *Shp* mRNA levels, which are direct FXR targets, were regulated similarly, and they correlated positively with small intestinal BA levels (*p* < 0.01, Spearman correlation coefficient [*r*_s_] = 0.517 and 0.520). Interestingly, when comparing WT and DKO mice fed an HFHSD for 47 weeks, ileal *Fgf15* expression levels were significantly lower in DKO mice, but serum FGF15 concentrations were significantly higher in DKO mice. This discrepancy suggests that FGF15 might be produced outside the ileum in DKO mice.

### HFHSD induces lipogenesis and inhibits fatty acid degradation in both genotypes

RNA-seq analysis after 30 weeks (Figure 5A) revealed that although genetic modification (DKO) did not markedly alter hepatic sugar, lipid, and amino acid metabolism, HFHSD induced glycolysis and lipogenesis while inhibiting fatty acid and amino acid degradation. HFHSD elevated liver tissue lipid levels (expressed per protein) mildly (Figure 5B). However, because HFHSD simultaneously increases liver weight and lipid and protein content, other methods (other than per mg of protein or g of liver) are needed to obtain a complete picture of lipid accumulation. To this end, we determined whole-liver lipid content, which confirmed a marked lipid increase (Supplemental Figure S5A, http://links.lww.com/HC9/B797).

The transcription factors, carbohydrate-responsive element-binding protein and SREBP1c, promote fatty acid biosynthesis. Insulin and LXR activate *Srebp1c* transcription, while *Chrebp* is transactivated by fructose, glucose, and LXR.^18^ While HFHSD markedly upregulated *Srebp1c* in both genotypes, it slightly increased *Chrebp* only at week 47 (Figure 5C and Supplemental Figure S5C, http://links.lww.com/HC9/B797). HFHSD upregulated acetyl-CoA carboxylase 1 (*Acc1*) and stearoyl-CoA desaturase 1 (*Scd1*), which are essential fatty acid biosynthesis enzymes (Figure 5D and Supplemental Figure S5D, http://links.lww.com/HC9/B797), and downregulated microsomal TG transfer protein (*Mttp*) (Figure 5E and Supplemental Figure S5E, http://links.lww.com/HC9/B797), which is negatively regulated by SREBPs.^19^

However, HFHSD downregulated the PPARα target genes, carnitine palmitoyltransferase 1 (*Cpt1*), acyl-CoA oxidase 1 (*Acox1*), and *Cyp4a10*, which are key enzymes in mitochondrial β-oxidation, peroxisomal β-oxidation, and microsomal ω-oxidation, respectively (Figure 5F and Supplemental Figure S5F, http://links.lww.com/HC9/B797). The liver levels of palmitoylcarnitine, a CPT1 product that facilitates β-oxidation by shuttling fatty acids into the mitochondria, were markedly elevated in DKO mice that were fed HFHSD for 47 weeks, probably because of simultaneous suppression of enzymes in the downstream β-oxidation pathway, including *Cpt2* (Supplemental Figure S5F, http://links.lww.com/HC9/B797). HFHSD-mediated fatty acid degradation suppression, especially in DKO mice, was confirmed by serum acetylcarnitine and 3-hydroxybutyrate concentrations, markers of systemic (muscle and liver) and liver-specific fatty acid β-oxidation, respectively (Figure 5H and Supplemental Figure S5H, http://links.lww.com/HC9/B797). In addition, HFHSD also decreased 3-hydroxyisobutyrate, an amino acid degradation marker,^20^ levels.

### Inflammation, and not metabolic abnormalities, causes oxidative stress in HFHSD-fed DKO mice

**FIGURE 6:**
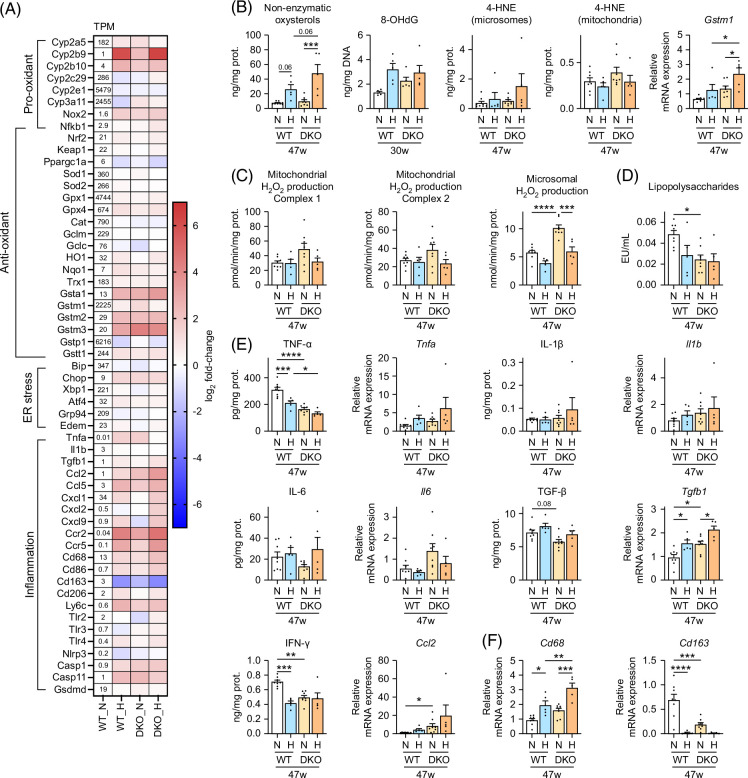
Liver oxidative stress, ER stress, and inflammation regulation in male WT and *Cyp2a12/Cyp2c70* DKO mice fed the indicated diets. (A) RNA sequencing was used to determine the relative mRNA levels of oxidative stress-, ER stress-, and inflammation-involved genes in WT versus DKO mice fed an ND or an HFHSD for 30 weeks (n = 3/group). The heatmap presents log 2-fold changes when compared with ND-treated WT mice. (B) Liver levels of oxidative stress markers and mRNA expression levels of *Gstm1* after 30 or 47 weeks. (C) Mitochondrial and microsomal H_2_O_2_ production after 47 weeks. Pyruvate + malate (Complex I) or succinate in the presence of rotenone (Complex II) was used as mitochondrial oxidative substrates, and NADPH, in the presence of SOD, was used as a microsomal oxidative substrate. (D) Serum LPS concentrations after 47 weeks. (E) Liver inflammatory cytokine concentrations and their mRNA levels after 47 weeks. (F) Liver pan-macrophage marker, CD68, and M2 macrophage marker, CD163, mRNA levels after 47 weeks. Data are presented as mean ± SEM. *, **, ***, and **** indicate *p* < 0.05, <0.01, <0.001, and <0.0001, respectively, based on 1-way ANOVA with a post hoc Tukey-Kramer test or Kruskal-Wallis with a post hoc Dunn-Bonferroni test. Abbreviations: 4-HNE, 4-hydroxynonenal; *Ccl2*, chemokine (C-C motif) ligand 2; *Cd*, cluster of differentiation; DKO, double knockout; ER, endoplasmic reticulum; *Gstm1*, glutathione S-transferase µ1; H, HFHSD; HFHSD, high-fat/high-sucrose diet; IFN-γ, interferon γ; LPS, lipopolysaccharide; N, ND; ND, normal diet; 8-OHdG, 8-hydroxydeoxyguanosine; SOD, superoxide dismutase; TPM, transcripts per million; WT, wild-type.

Figure 6A compares the mRNA levels of oxidative stress, endoplasmic reticulum stress, and inflammation genes at 30 weeks. Although HFHSD induced some pro-oxidant genes, it suppressed *Cyp3a11* and *Cyp2e1*, the main ROS sources that are negatively regulated by LXRα.^17^ Of the antioxidant genes, although there was no apparent change in NF-E2–related factor 2 target gene expression, genetic modification (DKO) and HFHSD markedly upregulated glutathione S-transferase α and µ (GSTα and µ), which are NF-E2–related factor 2 and LXRα target genes.^17^ Although endoplasmic reticulum stress gene changes were mild, DKO and HFHSD induced many inflammation-associated genes.

In both genotypes, HFHSD increased the levels of liver non-enzymatically produced oxysterols and 8-OHdG, which are oxidative stress markers (Figure 6B, Supplemental Figure S6A, http://links.lww.com/HC9/B797 and Supplemental Table S5, http://links.lww.com/HC9/B797), although it did not significantly affect 4-HNE, another oxidative stress marker, probably because of the induction of the 4-HNE-detoxifying enzyme, glutathione S-transferase.^21^ To determine if the metabolic abnormalities associated with DKO mice or HFHSD enhance ROS production, we directly measured hydrogen peroxide (H_2_O_2_) production by mitochondria or microsomes (Figure 6C and Supplemental Figure S6B, http://links.lww.com/HC9/B797). This analysis revealed that mitochondrial H_2_O_2_ production did not differ across the groups. However, H_2_O_2_ production in microsomes was significantly increased in DKO mice, but it reduced upon HFHSD treatment.

We then evaluated whether genetic modification (DKO) or HFHSD increased inflammation-derived ROS production. Serum levels of LPS, derived primarily from intestinal bacteria and trigger inflammatory cytokines release by macrophages, were not significantly elevated in DKO mice treated with HFHSD (Figure 6D and Supplemental Figure S6C, http://links.lww.com/HC9/B797). Liver TNF-α levels reflected changes in serum LPS concentrations (Figure 6E and Supplemental Figure S6D, http://links.lww.com/HC9/B797). However, unlike serum LPS levels, liver *Tnfa*, *Il1b*, *Il6*, C-C motif chemokine ligand 2 (*Ccl2*), and *Tgfb1* mRNA levels tended to be increased in DKO mice with HFHSD. Furthermore, while changes in the expression levels of these hepatic cytokines were consistent with liver expression levels of *Cd68*, a pan-macrophage marker, they were contrary to the levels of *Cd163*, an M2 macrophage marker (Figure 6F and Supplemental Figure S6E, http://links.lww.com/HC9/B797).

### HFHSD and CDCA synergistically increase HCC risk

**FIGURE 7:**
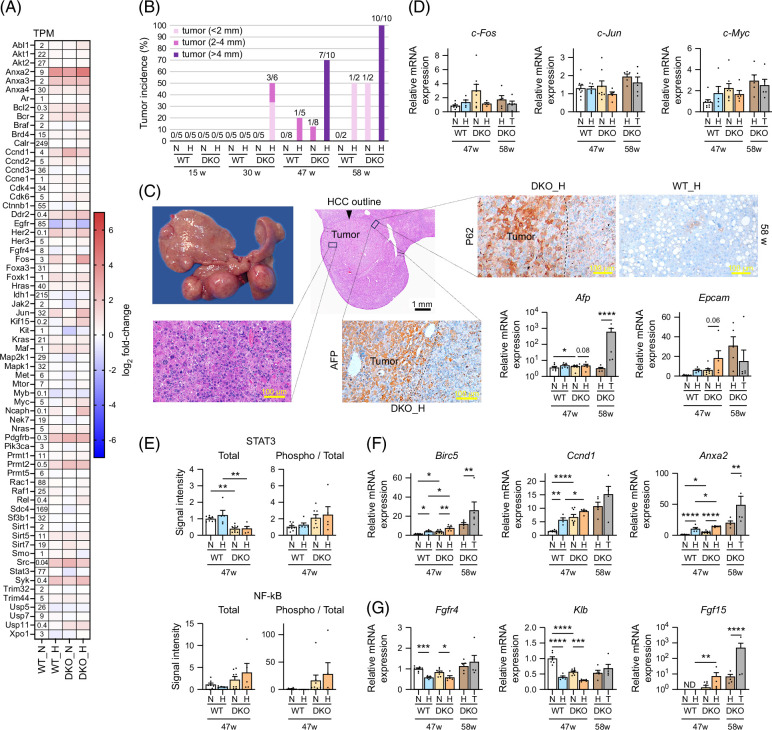
Liver HCC-related oncogene regulation in male WT and *Cyp2a12/Cyp2c70* DKO mice fed the indicated diets. (A) RNA sequencing was used to determine the relative mRNA levels of HCC-related oncogenes in WT versus DKO mice fed an ND or an HFHSD for 30 weeks (n = 3/group). The heatmap presents log 2-fold changes when compared with ND-treated WT mice. (B) Longitudinal tracking of tumor incidence. The fraction indicates the number of mice with tumor(s)/the number of examined mice. (C) Representative gross and microscopic images of liver tumors from DKO mice fed the HFHSD for 58 weeks. Immunohistochemical staining for liver AFP and P62, and the liver mRNA levels of tumor markers are shown. (D) Liver immediate early genes mRNA levels after 47 and 58 weeks. (E) Oncogenic transcription factor activation analysis after 47 weeks. (F) Liver mRNA levels of STAT3 target genes, and (G) FGF15-related genes after 47 and 58 weeks. Data are presented as mean ± SEM. *, **, ***, and **** indicate *p* < 0.05, <0.01, <0.001, and <0.0001, respectively, based on 1-way ANOVA with a post hoc Tukey-Kramer test or Kruskal-Wallis with a post hoc Dunn-Bonferroni test. Tumor and nontumor areas were compared after 58 weeks using *t* tests. Abbreviations: AFP, α-fetoprotein; *Birc5*, baculoviral IAP repeat containing 5; *Ccnd1*, cyclin D1; DKO, double knockout; *Epcam*, epithelial cell adhesion molecule; *Fgfr4*, FGF receptor 4; H, HFHSD; HFHSD, high-fat/high-sucrose diet; *Klb*, beta-Klotho; N, ND; ND, normal diet; ND, not detected, P62, sequestosome 1; STAT3, signal transducer and activator of transcription 3; T, tumor; TPM, transcripts per million; WT, wild-type.

RNA-seq analysis in nontumor tissues revealed that genetic modification (DKO) and HFHSD had similar effects on HCC-associated oncogenes (Figure 7A). At 30 weeks, HFHSD-fed DKO mice had a tumor incidence of 50%, which increased to 70% and 100% at 47 and 58 weeks, respectively (Figure 7B). The tumors in HFHSD-fed DKO mice were HCC, without fatty changes, and immunostaining and mRNA expression analysis revealed a marked increase in α-fetoprotein levels (Figure 7C). In nontumor tissues of HFHSD-fed DKO mice, the expression levels of the tumor-initiating cell marker, epithelial cell adhesion molecule (*Epcam*), increased with age, but they were not higher in tumor tissues. However, p62, a marker of HCC-initiating cells and impaired autophagy,^22^ was increased in HFHSD-fed DKO mice and markedly accumulated in tumor tissues (Figure 7C).

Although *c-Fos*, *c-Jun*, and *c-Myc* expression levels were not elevated in tumor tissues, *c-Fos* and *c-Jun* were transiently upregulated in some nontumor tissues from HFHSD-fed DKO mice at week 30 (Figure 7D and Supplemental Figure S7A, http://links.lww.com/HC9/B797). Analysis of the oncogenic transcription factors, STAT3 and NF-kB, activation (Figure 7E) revealed that the phosphorylation of STAT3 (Tyr705) and NF-kB P65 (Ser536) tended to increase in HFHSD-fed DKO mice, although not statistically significant. In addition, HFHSD treatment significantly upregulated the levels of the STAT3 target genes, survivin (*Birc5*), cyclin D1 (*Ccnd1*), and annexin A2 (*Anxa2*), which are antiapoptosis, proliferative, and hepatocyte pyroptosis^23^ factors, respectively, and the effect was more pronounced in DKO mice (Figure 7F).

IL-6 and FGF15 are known to activate STAT3. In DKO mice treated with HFHSD for 47 weeks, the expression and levels of liver IL-6 were not increased (Figure 6E), whereas serum FGF15 levels were significantly increased (Figure 4F). Because these serum FGF15 levels were disproportionately high when compared with ileal *Fgf15* levels, we suspected that in DKO mice, FGF15 was liver- or HCC-produced. Indeed, we observed that along with fibroblast growth factor receptor 4 (*Fgfr4*) and β-klotho (*Klb*), *Fgf15* was significantly expressed in the liver and some HCCs from DKO mice (Figure 7G and Supplemental Figure S8, http://links.lww.com/HC9/B797).

## DISCUSSION

The HFHSD-treated *Cyp2a12*^*–/–*^ *Cyp2c70*^*–/–*^ (DKO) mouse developed in this study is an important model for understanding human MASH and MASH-derived HCC pathogeneses. In this study, we chose to focus on male mice, considering the comparisons with previously reported MASH models. Female DKO mice are at higher risk of liver injury due to factors other than HFHSD,^9^ which is another reason for using male mice. When compared with traditional models,^6^,^7^ this model replicates disease progression in conditions that resemble the human Western diet more closely. Specifically, carcinogens, or excess cholesterol or fructose, were not administered. Humans lack the genes that we knocked out, and artificially modified human orthologs were not made. Nevertheless, this model progressed to MASH with obesity and insulin resistance, and eventually, it developed HCC after 30 weeks or more. Moreover, at 58 weeks, the model reproduced advanced-stage human MASH, with disappeared steatosis, which is referred to as burned-out MASH.

A comparison of HFHSD-treated DKO and WT mice revealed relatively milder insulin resistance and liver steatosis in DKO mice. In DKO mice, FXR activation by hydrophobic BAs may improve insulin resistance.^24^ Takeda G protein–coupled receptor 5 activation by secondary BAs is reported to prevent obesity and insulin resistance through energy expenditure induction in brown adipose tissue and skeletal muscles,^25^ as well as by stimulating GLP-1 secretion from intestinal endocrine cells.^26^ However, the body weight of DKO versus WT mice was not significantly different, and total fasting serum (active and inactive) GLP-1 levels were not elevated in DKO mice. The lack of basal GLP-1 secretion increase despite higher secondary BA concentrations in DKO mice is probably because of activated FXR counter-effects on GLP-1 secretion.^27^

Although DKO mice have favorable steatosis and insulin resistance features when compared with WT mice, there was more HFHSD-mediated fibrosis in DKO mice (Figure 2C), and as early as after 15 weeks, fibrosis was observed in DKO mice only. However, at 15 weeks, inflammation (based on histology and liver cytokine assays) and oxidative stress (based on liver non-enzymatic oxysterols) levels did not differ significantly in DKO versus WT mice. A recent study reported that TCDCA activates mouse HSCs by enhancing the mitochondrial permeability transition Caspase-11 pyroptosis pathway in hepatocytes and HSCs.^28^ This direct TCDCA action on HSCs may explain liver fibrosis induction in DKO mice without inflammation or oxidative stress differences when compared with WT mice. However, after 47 weeks of the HFHSD, hepatic inflammation and oxidative stress became more apparent in DKO mice than in WT mice, probably because of lipotoxicity’s (eg, accumulated free cholesterol and saturated FFA) synergistic effects^29^ and further liver TCDCA level elevation. Although LPS promotes MASH development,^30^ DKO mice did not have higher serum LPS levels than WT mice, which were rather lower at 47 weeks. Serum LPS levels correlated well with hepatic TNFα levels but not with hepatic *Tnfa* mRNA expression (Figure 6E). Since liver cytokine levels are affected by cytokines produced outside the liver, gene expression may be a better way of evaluating liver-derived cytokine production.

Increased TCDCA and toxic lipid levels in the livers of HFHSD-fed DKO mice were associated with this model’s characteristic metabolic abnormalities. At least 3 mechanisms may increase hepatic TCDCA levels in HFHSD-fed, aged DKO mice. First, CYP8B1, but not CYP7A1, was markedly downregulated (Figure 4B), probably because of FXR and LXRα coactivation. Second, through gut microbiota alteration, HFHSD reduces BA deconjugation and 7α-dehydroxylation.^31^ Third, while intrahepatic cholestasis progressed with liver injury, hepatic BA levels increased. Moreover, CYP8B1 downregulation, but not CYP7A1,^32^ decrease in the serum levels of unconjugated BAs and secondary BAs,^33^ and hepatic BA increase,^34^ have been reported in patients with MASH.

However, when fed HFHSD, both mouse genotypes exhibited liver toxic-lipid accumulation. HFHSD-associated liver cholesterol increase was probably because of an influx of extrahepatically synthesized cholesterol into the liver. Indeed, HFHSD downregulated hepatic enzymes in the cholesterol biosynthetic pathway with increasing serum cholesterol levels (mainly HDL) and upregulating hepatic scavenger receptor class B type 1 (*Scarb1*) (Figure 4A). Hepatic TG and FFA accumulation during HFHSD were caused by increased FFA uptake and de novo lipogenesis and decreased TG export and FFA β- and ω-oxidation levels. These pathophysiologies are similar to those observed in human MASLD.^35^ Human MASH is characterized by PPARα target gene downregulation,^36^ which was also observed in our HFHSD model. Because LXRα and PPARα compete for the heterodimeric partner, retinoid X receptor α, strong LXRα activation suppresses PPARα.^37^

Although FFA oxidation is the main cause of ROS production, this was not the case in our HFHSD mouse model. In the HFHSD-fed mice, increased ROS production seemed to result from LPS-driven inflammation, lipotoxicity, and TCDCA-induced liver injury, although they depended on genotype and age. ROS-induced oxidative DNA damage is strongly suggested to cause carcinogenesis.^38^,^39^ However, the markedly higher HCC incidence in HFHSD-fed DKO mice is challenging to explain based on ROS level differences only (Figure 6B). Since the target genes of the oncogenic transcription factor, STAT3, were upregulated in tumor and nontumor tissues from DKO-HFHSD mice, we speculated that in HFHSD-fed DKO mice, STAT3 was activated before hepatocarcinogenesis. Various cytokines and growth factors, including IL-6 and FGF15/19,^40^,^41^ activate (phosphorylate) STAT3, and aged HFHSD-fed DKO mice exhibited more severe liver inflammation and the highest serum FGF15 levels compared with the other mice. Unlike in humans, it is widely believed that in mice, *Fgf15* is expressed in small intestines only, and not in the liver.^42^ However, we observed significant liver *Fgf15* expression in aging DKO mice. Human FGF19, which is thought to promote hepatocyte proliferation, may lead to carcinogenesis,^43^,^44^ while in mice, there are positive^45^ and negative^43^ reports of FGF15 promoting carcinogenesis. Our results suggest that, at least in the presence of hydrophobic and cytotoxic BAs, FGF15 may promote hepatocarcinogenesis.

Hepatic BA composition promotes hepatic inflammation, fibrosis, and carcinogenesis. In WT mice with hydrophilic BA composition, HFHSD caused more severe hepatic steatosis than in DKO mice, although inflammation, fibrosis, and carcinogenesis were inhibited. While the treatment of human MASH using hydrophilic ursodeoxycholic acid has been tried, no significant effects were reported on anthropometric characteristics or liver histology,^46^ although it may induce liver-neutral lipid accumulation.^47^ Nevertheless, based on our results, hydrophilic BAs may help slow fibrosis and prevent HCC development.

Although our mouse model mimics many human pathology aspects successfully, species differences remain. For example, while CDCA often causes liver injury in humans,^48^ when compared with human hepatocytes, mouse hepatocytes seem more sensitive to CDCA-induced damage. While taurine is the amino acid most commonly conjugated to BAs in mice, in humans, glycine conjugation is more abundant. However, the mitochondrial permeability transition caspase-11 pyroptosis pathway seems to be activated by glycine-conjugated CDCA and TCDCA.^28^ Because the promoter of human *CYP7A1* lacks an LXRα binding site, LXRα upregulates mouse *Cyp7a1* but not the human gene.^49^ In addition, after HFHSD administration, mice had significantly lower serum TG levels, which was associated with LXRα activation–mediated lipoprotein lipase upregulation (Figure 5A) and MTTP downregulation.^50^ Therefore, these species differences require careful consideration during results interpretation and in future studies.

**FIGURE 8:**
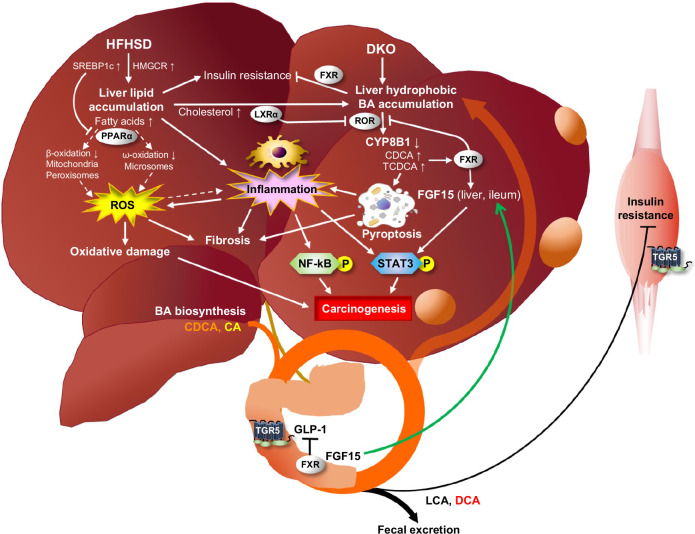
Schematic representation of this study. In this model, in *Cyp2a12/Cyp2c70* DKO mice, HFHSD and human-like hydrophobic BAs are essential for the development of metabolic dysfunction–associated steatotic liver disease (MASH) and HCC. In HFHSD, sucrose induces SREBP1c, which downregulates fatty acid oxidation through PPARα inhibition, resulting in liver lipid accumulation and lipotoxicity. Increased cholesterol synthesis and activated LXRα upregulate CYP7A1 and downregulate CYP8B1, thereby promoting liver CDCAs synthesis and accumulation. In addition to lipotoxicity, the accumulated CDCAs promote fibrosis and carcinogenesis through pyroptosis, inflammation, oxidative stress, and liver FGF15 expression. Abbreviations: BA, bile acid; CDCA, chenodeoxycholic acid; DKO, double knockout; HFHSD, high-fat/high-sucrose diet; LXRα, liver X receptor α; MASH, metabolic dysfunction–associated steatohepatitis; PPARα, peroxisome proliferator–activated receptor α; SREBP1c, sterol regulatory element-binding protein 1c.

In conclusion, this study generated a mouse model of MASH with HCC in conditions similar to those occurring in humans. This model indicates that HFHSD and hydrophobic human BA composition, especially CDCA, play essential roles in MASH and HCC pathogeneses (Figure 8).

## Supplementary Material

SUPPLEMENTARY MATERIAL
