# Supplementary material for: High-fat/high-sucrose diet results in a high rate of MASH with HCC in a mouse model of human-like bile acid composition
Source: Hepatol Commun. 2024 Dec 11;9(1):e0606. doi: 10.1097/HC9.0000000000000606 (PMC11637755; doi:10.1097/HC9.0000000000000606)
Supplement: SUPPLEMENTARY MATERIAL [file hc9-9-e0606-s001.pdf]

High-fat/high-sucrose diet results in a high rate of MASH with HCC in a mouse model of human-like bile acid composition

Hajime Ueda, Akira Honda, Teruo Miyazaki, Yukio Morishita, Takeshi Hirayama, Junichi Iwamoto, Tadashi Ikegami

Table of contents

Supplemental materials and methods······························2

Supplemental tables S1 – S6··8

Supplemental figures S1 – S8··15

SUPPLEMENTAL MATERIALS AND METHODS

Measurement of liver ROS production

Amplex Red Hydrogen Peroxide/Peroxidase Assay Kit and Amplex Red/UltraRed Stop Reagent were obtained from Thermo Fisher Scientific (Waltham, MA). Superoxide dismutase (SOD), from bovine erythrocytes (Cu/Zn type) was purchased from FUJIFILM Wako Pure Chemical (Osaka, Japan). Mitochondria and microsomes were prepared from livers by differential ultracentrifugation.[1]

Measuring mitochondrial H_2O_2 production

The rate of H_2O_2 production was measured following the oxidation of Amplex Red by horseradish peroxidase as reported previously.[2,3] Approximately 150 µg protein of mitochondria were washed with a HEPES-phosphate buffer (pH 7.4) consisting of 0.1 mM EGTA, 5 mM KH_2PO_4, 3 mM $MgCl_2$, 145 mM KCl, and 30 mM HEPES (to eliminate DTT). After centrifugation at 20,000 *g* for 1 min, the supernatant was discarded, and the pellet was resuspended in 50 µl of the HEPES-phosphate buffer (pH 7.4). Then, 50 µl of the following Complex I or Complex II assay buffer was added and incubated at 37°C.

Complex I assay buffer: HEPES-phosphate buffer (pH 7.4) containing 10 mM pyruvate, 5 mM malate, 0.6% (w/v) BSA, 0.1 mM Amplex Red reagent, and 0.2 U/ml HRP.

Complex II assay buffer: HEPES-phosphate buffer (pH 7.4) containing 10 mM succinate, 8 µM rotenone, 0.6% (w/v) BSA, 0.1 mM Amplex Red reagent, and 0.2 U/ml HRP.

After incubation for 5 min, 20 µl of Amplex Red Stop Reagent was added and centrifuged at 20,000 *g* for 1 min. The fluorescence of supernatant was measured using 570 nm as the excitation wavelength and 600 nm as the emission wavelength. The rate of H_2O_2 production was calculated using a standard curve of H_2O_2.

Measuring microsomal H_2O_2 production

We measured the rate of H_2O_2 production in microsomes according to the previous report.[4] Four µl of microsomal fraction (ca. 100 µg protein) was added to 96 µl of HEPES-phosphate buffer (pH 7.4) consisting of 0.1 mM EGTA, 5 mM KH_2PO_4, 3 mM $MgCl_2$, 145 mM KCl, and 30 mM HEPES. After gentle mixing, 5 µl of the solution (ca. 5 µg protein) was transferred into a new tube, to which 35 µl of the HEPES-phosphate buffer (pH 7.4) and 50 µl of the following microsomal assay mixture was added and preincubated at 37°C for 3 min.

Microsomal assay mixture: HEPES-phosphate buffer (pH 7.4) containing 200 U/ml SOD, 0.1 mM Amplex Red reagent, and 0.2 U/ml HRP.

Then, 10 µl of 2 mM NADPH in the HEPES-phosphate buffer was added and

incubated at 37°C for 10 min. The reaction was stopped by adding 20 µl of Amplex Red Stop Reagent. After centrifugation at 20,000 *g* for 1 min, the fluorescence of supernatant was measured using 570 nm as the excitation wavelength and 600 nm as the emission wavelength. The rate of H_2O_2 production was calculated using a standard curve of H_2O_2.

1. Honda A, Salen G, Matsuzaki Y, et al. Differences in hepatic levels of intermediates in bile acid biosynthesis between Cyp27$^{-/-}$ mice and CTX. J Lipid Res. 2001;42:291–300.
2. Serviddio G, Sastre J. Measurement of mitochondrial membrane potential and proton leak. Methods Mol Biol. 2010;594:107–21.
3. Bellanti F, Romano AD, Giudetti AM, et al. Many faces of mitochondrial uncoupling during age: damage or defense? J Gerontol A Biol Sci Med Sci. 2013;68:892–902.
4. Mishin V, Heck DE, Laskin DL, Laskin JD. The Amplex Red/horseradish peroxidase assay requires superoxide dismutase to measure hydrogen peroxide in the presence of NAD(P)H. Free Radic Res. 2020;54:620-8.

Quantifying liver 8-hydroxydeoxyguanosine (8-OHdG)

Liver 8-OHdG concentrations were measured by an ELISA kit (Highly sensitive 8-OHdG Check, Japan Institute for the Control of Aging, Shizuoka, Japan) using 50 µg of total genomic DNA extracted and hydrolyzed by DNA Extractor TIS Kit and 8-OHdG Assay Preparation Reagent Set (FUJIFILM Wako), respectively.

Quantifying liver 4-hydroxynonenal (4-HNE)

4-HNE was obtained from Sigma-Aldrich (St. Louis, MO) and [$^2H_3$]4-HNE was from Santa Cruz Biotechnology (Dallas, TX). Girard's reagent P was purchased from Tokyo Chemical Industry (Tokyo, Japan). Additional reagents and solvents were of analytical grade.

[$^2H_3$]4-HNE (0.5 ng) in 10 µl of ethanol as an internal standard was added to liver microsomes (0.2 mg protein/10 µl) or mitochondria (0.15 mg protein/10 µl). After adding 170 µl of ethanol, the mixture was vortexed for 1 min and centrifuged at 20,000 *g* for 1 min. The supernatant was collected, and 20 µl of acetic acid and 10 µl of freshly prepared GP reagent (2 mg of Girard's reagent P/ml H_2O) were added. After heating at 60°C for 10 min, the solvent was evaporated at 60°C under a nitrogen stream. The residue was redissolved in 100 µl of acetonitrile, and an aliquot (5 µl) was injected into the following LC-MS/MS system.

The LC-MS/MS system consisted of a TSQ Vantage triple stage quadrupole mass spectrometer (Thermo Fisher Scientific) equipped with an HESI-II probe and a Prominence ultra-fast liquid chromatography (UFLC) system (Shimadzu, Kyoto, Japan). Chromatographic separation was performed using a Hypersil GOLD column (150 x 2.1

mm, 3 µm, Thermo Fisher Scientific) at 40°C, and the following gradient system was used at a flow rate of 300 µl/min: initially, the mobile phase was comprised of acetonitrile–H_2O (5:95, v/v) containing 0.2% formic acid; then it was programmed in a linear manner to acetonitrile–water (1:1, v/v) containing 0.2% formic acid over 20 min. Finally, the column was washed with 100% acetonitrile with 0.2% formic acid for 5 min. The general MS/MS conditions were as follows: spray voltage, 3,000 V; vaporizer temperature, 350°C; sheath gas (nitrogen) pressure, 55 psi; auxiliary gas (nitrogen) flow, 25 arbitrary units; ion transfer capillary temperature, 300°C; collision gas (argon) pressure, 1.0 mTorr; and ion polarity, positive. Selected reaction monitoring (SRM) was conducted using *m/z* 290 → *m/z* 120 (CE: 25 V) for the 4-HNE derivatized with GP reagent and *m/z* 293 → *m/z* 120 (CE: 25 V) for the $^2H_3$ variant.

Quantification of serum organic compounds

Sodium D-3-hydroxybutyrate (3-HB), sodium 3-hydroxyisobutyrate (3-HIB), and acetyl-L-carnitine HCl were purchased from Sigma-Aldrich (St. Louis, MO), L-carnitine, choline chloride, and taurine were from FUJIFILM Wako Pure Chemical, and trimethylamine N-oxide (TMAO) anhydrous was from Tokyo Chemical Industry (Tokyo, Japan). Sodium DL-[$^{13}C_4$]3-HB was obtained from Taiyo Nippon Sanso (Tokyo, Japan) and DL-[$^2H_9$]carnitine HCl, acetyl-L-[$^2H_3$]carnitine HCl, [$^2H_9$]choline HCl, and [$^2H_4$]taurine were from C/D/N Isotopes (Quebec, Canada). Additional reagents and solvents were of analytical grade.

An internal standard mixture of DL-[$^{13}C_4$]3-HB (100 ng), [$^2H_9$]carnitine (25 ng), acetyl-L-[$^2H_3$]carnitine (12.5 ng), [$^2H_9$]choline (100 ng), and [$^2H_4$]taurine (100 ng) in 100 µl of acetonitrile–0.1% formic acid in H_2O (95:5, v/v) was added to 5 µl of serum, vortexed for 30 seconds, and centrifuged at 20,000 *g* for 1 min. The supernatant was collected, and the solvent was evaporated at 60°C under a nitrogen stream. The residue was redissolved in 70 µl of H_2O containing 0.1% formic acid, and an aliquot (5 µl) was injected into the following LC-MS/MS system.

Chromatographic separation was performed using a Hypersil GOLD aQ column (150 x 2.1 mm, 3 µm, Thermo Fisher Scientific) at 40°C. The mobile phase consisted of methanol–H_2O (1:9, v/v) containing 0.1% formic acid and was used at a flow rate of 200 µl/min. Polarity switching mode was employed in mass spectrometry. The general MS/MS conditions were as follows: vaporizer temperature, 300°C; sheath gas (nitrogen) pressure, 50 psi; auxiliary gas (nitrogen) flow, 20 arbitrary units; ion transfer capillary temperature, 300°C; and collision gas (argon) pressure, 1.0 mTorr. For positive ion mode, the spray voltage was 3,000 V, and SRM was conducted using *m/z* 103 → *m/z* 59 (CE: 15 V) for 3-HB, *m/z* 103 → *m/z* 73 (CE: 15 V) for 3-HIB, *m/z* 124 → *m/z* 80 (CE: 20 V) for taurine, *m/z* 107 → *m/z* 61 (CE: 15 V) for [$^{13}C_4$]3-HB, and *m/z* 128 → *m/z* 80 (CE: 20 V) for [$^2H_4$]taurine. [$^{13}C_4$]3-HB was also used as an internal standard for 3HIB. For negative ion mode, the spray voltage was 2,000 V, and SRM was conducted using *m/z* 76 → *m/z* 59 (CE: 13 V) for TMAO, *m/z* 104 → *m/z* 60 (CE: 20 V)

for choline, *m/z* 162 → *m/z* 103 (CE: 20 V) for carnitine, *m/z* 204 → *m/z* 85 (CE: 20 V) for acetylcarnitine, *m/z* 113 → *m/z* 69 (CE: 20 V) for [$^2H_9$]choline, *m/z* 171 → *m/z* 103 (CE: 20 V) for [$^2H_9$]carnitine and *m/z* 207 → *m/z* 85 (CE: 20 V) for [$^2H_3$]acetylcarnitine. [$^2H_9$]choline was also used as an internal standard for TMAO.

Quantification of liver palmitoylcarnitine

Palmitoyl-L-carnitine HCl was purchased from Sigma-Aldrich (St. Louis, MO) and palmitoyl-L-[$^2H_3$]carnitine HCl was obtained from C/D/N Isotopes (Quebec, Canada). Additional reagents and solvents were of analytical grade.

Five µl of 1 µM [$^2H_3$]palmitoylcarnitine in acetonitrile–0.1% formic acid in H_2O (95:5, v/v) as an internal standard was added to 5 µl of liver homogenate (1 mg equivalent of liver). After adding 50 µl of 0.1% formic acid in methanol, the mixture was vortexed for 1 min and centrifuged at 20,000 *g* for 1 min. The supernatant was collected, and an aliquot (2 µl) was injected into the following LC-MS/MS system.

Chromatographic separation was performed using a Hypersil GOLD aQ column (150 x 2.1 mm, 3 µm) at 40°C. The mobile phase consisted of methanol–H_2O (9:1, v/v) containing 0.1% formic acid and was used at a flow rate of 300 µl/min. The general MS/MS conditions were as follows: spray voltage, 3,000 V; vaporizer temperature, 350°C; sheath gas (nitrogen) pressure, 55 psi; auxiliary gas (nitrogen) flow, 25 arbitrary units; ion transfer capillary temperature, 300°C; collision gas (argon) pressure, 1.0 mTorr; and ion polarity, positive. Selected reaction monitoring (SRM) was conducted using *m/z* 400 → *m/z* 85 (CE: 20 V) for Palmitoylcarnitine and *m/z* 403 → *m/z* 85 (CE: 20 V) for the $^2H_3$ variant.

Histopathological examinations

Liver samples were fixed in 10% neutral buffered formalin and embedded in paraffin blocks. Each paraffin block was cut into 4 µm sections and stained using hematoxylin/eosin and Masson's trichrome.

An expert liver pathologist evaluated liver histopathology, and the severity of steatosis, lobular inflammation, and hepatocellular ballooning was scored based on the NASH-Clinical Research Network scoring system.[5] Specifically, the amount of steatosis (percentage of hepatocytes with fat droplets) was scored as 0 (<5%), 1 (5–33%), 2 (>33–66%), or 3 (>66%). Hepatocyte ballooning was classified as 0 (none), 1 (few balloon cells), or 2 (many cells/prominent ballooning). Lobular inflammation was scored as 0 (no inflammatory foci), 1 (<2 foci per 200× field), 2 (2–4 foci per 200× field), or 3 (>4 foci per 200× field). Fibrosis was scored as 0 (none), 1 (perisinusoidal or periportal), 2 (perisinusoidal and portal/periportal), 3 (bridging fibrosis), or 4 (cirrhosis). Steatohepatitis was diagnosed according to the FLIP algorithm using steatosis, ballooning, and lobular inflammation.[6]

Immunohistochemical (IHC) stain was carried out using an auto-stain system with the exclusive reagents (Discovery XT system, VENTANA, Roche Diagnostics K.K., Basel, Switzerland) and M.O.M. (Mouse on Mouse) Immunodetection Kit (Vector Laboratories, Burlingame, CA).[7] Deparaffinized 4 µm thick specimens of liver tissue were heated with Tris-EDTA buffer pH 7.8 at 95°C for 30 minutes for antigen retrieval and then were blocked with Mouse IgG Blocking Reagent and Protein Concentrate solutions (M.O.M.) for 1 hour and 5 minutes, respectively. Then, the specimens were incubated with each of the primary polyclonal antibodies of Alpha-1-Fetoprotein (1:100; ACR 028 A, Biocare Medical, Pacheco, CA) and P62, SQSTM1 (1:100; 18420-1-AP, Proteintech, Rosemont, IL) for 32 min at 37ºC. Thereafter, the incubations with secondary antibody and detection were developed using the VENTANA ultraView universal DAB detection kit (VENTANA), and the nucleus and cytoplasm were stained by the Hematoxylin II Counterstain (VENTANA) and the bluing reagent (VENTANA), respectively. Specific immunoreaction of the primary antibodies was confirmed by incubation without each antibody.

IHC stain for hepatic and intestinal Fgf15 expression was performed by GenoStaff (Tokyo, Japan). The tissue sections on slides were deparaffinized and antigen retrieval was performed using citrate buffer (pH 6.0) and microwave followed by incubating in 0.3% H_2O_2 in methanol to block endogenous peroxidase. After treated with blocking reagent (G-Block, GenoStaff), the sections were incubated overnight with with 2 μg/ml of polyclonal sheep anti-Fgf15 antibody (AF6755, R&D Systems, Minneapolis, MN). Rabbit Anti-Sheep IgG (ab6746, Abcam, Cambridge, UK) was used as a secondary antibody, according to the manufacturer's instructions.

Oil Red O stains were performed using Lipid Assay Kit (Cosmo Bio, Tokyo, Japan).

5. Kleiner DE, Brunt EM, Natta MV, et al. Design and validation of histological scoring system for nonalcoholic fatty liver disease. Hepatology. 2005;41:1313-21.
6. Bedossa P, Consortium FP. Utility and appropriateness of the fatty liver inhibition of progression (FLIP) algorithm and steatosis, activity, and fibrosis (SAF) score in the evaluation of biopsies of nonalcoholic fatty liver disease. Hepatology 2014;60:565-75.
7. Miyazaki T, Doy M, Unno R et al. Regulatory T cells and liver pathology in a murine graft versus host response model. Hepatol Res. 2009;39:585-94.

RNA-seq analysis

RNA-seq analysis was performed by TaKaRa Bio, Inc. (Kusatsu, Japan). Total liver RNA from each mouse (n = 3 per group) was used for mRNA amplification by 5' template switching PCR using the SMART-Seq v4 Ultra Low Input RNA Kit (TaKaRa). The cDNA was fragmented and appended with dual-indexed barcodes using Illumina Nextera XT DNA Library Prep Kits. The libraries were validated on an Agilent 4200 TapeStation, pooled, and sequenced on an Illumina NovaSeq 6000.

ELISA assays

Item	Source	Catalog #
Mouse FGF15 ELISA Kit	MyBioSource (San Diego, CA)	MBS2700661
Mouse/Rat FGF-21 ELISA Kit	Proteintech	KE10042
Total GLP-1-HS ELISA Kit	Yanaihara Institute (Fujinomiya, Japan)	YK161
Mouse/Rat adiponectin ELISA Kit	Otsuka Pharmaceutical Co. (Tokyo, Japan)	410713
LBIS Mouse TNF-α ELISA Kit	FUJIFILM Wako Pure Chemical	634-44721
Mouse IL-1β ELISA Kit	RayBiotech (Norcross, GA)	ELM-IL1b
Mouse IL-6 Assay Kit - IBL	Immuno-Biological Laboratories Co. (Fujioka, Japan)	27768
Mouse TGF-β1 ELISA Kit	Proteintech	KE10005
LBIS Mouse IFN-γ ELISA Kit	FUJIFILM Wako Pure Chemical	630-44701
Phosphatase Inhibitor Cocktail (100x)	Nacalai Tesque (Kyoto, Japan)	07574-61
Phospho-Stat 3 (Y705) and Total Stat 3 ELISA Kit	RayBiotech	PEL-Stat3-Y705-T
Phospho-NF-KB p65 (S536) ELISA and Total NF-KB p65 Kit	RayBiotech	PEL-NFKBP65-S536-T

Supplemental Table S1. Nutritional contents of the normal chow diet (CRF-1) and high-fat/high-sucrose diet (F2HFHSD)

per 100 g	Chow (CRF-1)	HFHS (F2HFHSD)
General ingredients		
Moisture (g)	8.2	8.2
Crude protein (g)	21.9	21.7
Crude fat (g)	5.4 (cholesterol 65 mg)	28.7 (cholesterol 24 mg)
Crude ash (g)	6.3	3.0
Crude fiber (g)	2.9	4.8
Nitrogen free extracts (g)	55.3	33.6 (sucrose 20.0)
Calories		
Total calories (kcal)	357.4	479.4
From protein (%)	24.5	18.1
From fat (%)	13.6	53.9
From nitrogen free extracts (%)	61.9	28.0
Minerals		
Calcium (g)	1.22	0.50
Phosphorus (g)	0.81	0.35
Magnesium (g)	0.23	0.05
Sodium (g)	0.26	0.10
Potassium (g)	0.86	0.35
Iron (mg)	13.80	3.50
Copper (mg)	0.95	0.60
Zinc (mg)	6.15	3.01
Manganese (mg)	7.27	1.05
Vitamins		
Vitamin A (IU)	3245	400
Vitamin D (IU)	643	100
Vitamin E (mg)	20.3	7.50
Vitamin B1 (mg)	4.74	0.60
Vitamin B2 (mg)	3.31	0.60
Vitamin B6 (mg)	1.27	0.70
Niacin (mg)	15.6	3.00
Pantothenic acid (mg)	5.07	1.60
Choline (g)	0.26	0.10
Folate (mg)	0.27	0.20

This table was reproduced from our previous paper (Iwamoto et al. Hepatol Commun. 2021, 5: 2052-2067).

Supplemental Table S2. Sequences of oligonucleotide primers for qRT-PCR.

mRNA	Genbank Accession No.	Forward	Reverse	Amplicon Length (bp)
Hmgcr	NM_008255	5’-TGCCATCGATAGAGATAGGAA-3’	5’-GCCATCACAGTGCCACATA-3’	154
Fxr	NM_001163700	5’-GGTCATGCAGACCTGTTGGAA-3’	5’-TGACGATCGCTGTGAGCAGA-3’	142
Shp	NM_011850	5’-CAAGGAGTATGCGTACCTGA-3’	5’-GATAGGGCGGAAGAAGAGAT-3’	232
Cyp7a1	NM_007824	5’-AAGAGCAACTAAACAACCTG-3’	5’-TTCCCACTTTCATCAAGGTA-3’	244
Cyp8b1	NM_010012	5’-TCCTGAGCTTATTCGGCTACA-3’	5’-ATCGACGGAACTTCCTGAAC-3’	81
Cyp27a1	NM_024264	5’-CTTCCTGCTGACCAATGAAT-3’	5’-AGCTTTTAGCAGAGGCATGT-3’	229
Cyp7b1	NM_007825	5’-CAACATGGTGACACTTTCACTG-3’	5’-CTGATAATCGGCTGCTGAACT-3’	133
Ostb	NM_178933	5’-AACATGGACCACAGTGCAGAGA-3’	5’-GCTTGTCATGACCACCAGGA-3’	150
Fgf15	NM_008003	5’-GCGGACGGCAAGATATACGG-3’	5’-GGCCTGGATGAAGATGATATGGA-3’	132
Fgfr4	NM_008011	5’-CTGCTGGCTGTCTCTGAAGAGT-3’	5’-TGGCTGAAAACCGAGTCAC-3’	113
Klb	NM_031180	5’-GAGACAGGGATATCTACATCACAG-3’	5’-CAGTCAGTTTGAATGCATAGTAGC-3’	162
Asbt	NM_011388	5’-TATGGGTTGCTGCCCTGGA-3’	5’-GTGTGGAGCAAGTGGTCATGCTA-3’	98
Chrebp	NM_001359237	5’-ATCTGCAGATCGCGTGGAG-3’	5’-CGGATCTTGTCCCGGCATAG-3’	93
Srebp1c	NM_001358314	5’-ATCGGCGCGGAAGCTGTCGG-3’	5’-GAAGTCACTGTCTTGGTTGTTG-3’	103
Acc1	NM_133360	5’-GCTGCTGGAGGACTTCAT-3’	5’-CCAGCCAGTGTCGATTCT-3’	204
Scd1	NM_009127	5’-TGCTATCGGGGTGTTAATGA-3’	5’-TGTCTTGTGGCATGGTTA-3’	128
Abcg1	NM_009593	5’-CCGTGAACATCGAATTCAAGGA-3’	5’-CATGATGGCCACCAGCTCTC-3’	131
Cd36	NM_001159558	5’-CATATTGGTCAAGCCAGCTAGAA-3’	5’-TCACCAATGGTCCCAGTCTC-3’	102
Mttp	NM_001163457	5’-CATCTCCACAGTGCAGTTCTCACA-3’	5’-GGAGTTCACATCCGGCCACTA-3’	167
Cyp3a11	NM_007818	5’-GGCAGCATTGATCCTTATG-3’	5’-AAGAACTCCTTGAGGGAGAC-3’	260
Mdr2	NM_008830	5’-ACCAGTGTCTTTTCTGAAGGTC-3’	5’-CTGCTTCACTGCGTCATC-3’	166
Cpt1a	NM_013495	5’-GGGAGAGAATTTCATCCACT-3’	5’-TGGTTTGTATCACTAGAGTCCA-3’	189
Cpt2	NM_009949	5’-CTGACCAAAGAAGCAGCGAT-3’	5’-GGTATGCAGGGTCCTGATAGA-3’	115
Acox1	NM_015729	5’-GGCATTGGCATCGTGAGAAC-3’	5’-GCAAATCTGATGGCTTTGACTTGA-3’	96
Cyp4a10	NM_010011	5’-TCCAGCAGTTCCCATCACCT-3’	5’-TTGCTTCCCCAGAACCATCT-3’	136
Gstm1	NM_010358	5’-CAAGCTGGGCCTGGACTTTC-3’	5’-ACAATGTCTGCACGGATCCTC-3’	147
Il1b	NM_008361	5’-TCCAGGATGAGGACATGAGCAC-3’	5’-GAACGTCACACACCAGCAGGTTA-3’	105
Il6	NM_031168	5’-GTCGGAGGCTTAATTACACATGTTC-3’	5’-GCAAGTGCATCATCGTTGTTCA-3’	101
Tnfa	NM_001278601	5’-ACTCCAGGCGGTGCCTATGT-3’	5’-GTGAGGGTCTGGGCCATAGAA-3’	160
Ccl2	NM_011333	5’-CCACTCACCTGCTGCTACTCAT-3’	5’-TGGTGATCCTCTTGTAGCTCTCC-3’	76
Tgfb1	NM_011577	5’-TGGAGCAACATGTGGAACTC-3’	5’-GTCAGCAGCCGGTTACCA-3’	73
Cd4	NM_013488	5’-CTAGTTCCAGGCCCTCGGTA-3’	5’-GGGTGAGAACAGCAGTGATCAA-3’	136
Cd68	NM_001291058	5’-AGCATAGTTCTTTCTCCAGC-3’	5’-ATGATGAGAGGCAGCAAGAG-3’	137
Cd163	NM_001170395	5’-GTGGACTCTGAAGCGACGACA-3’	5’-AAGTCCAGATCATCCGCCTTTG-3’	114
F4/80	NM_010130	5’-AGCTGTAACCGGATGGCAAAC-3’	5’-GACTGTACCCACATGGCTGATGA-3’	96

Mpo	NM_010824	5’-AAGCGCCTGAATCCTCGATG-3’	5’-GCAGGTAGTCCCGGTATGTGATG-3’	100
Timp1	NM_001294280	5’-TCAAAGACCTATAGTGCTGGCTGTG-3’	5’-AAAGTGACGGCTCTGGTAGTCCTC-3’	141
Col1a1	NM_007742	5’-GGACCTCAGGGTATTGCTGGA-3’	5’-AAGGACCTTGTTTGCCAGGTTC-3’	115
Afp	NM_007423	5’-ACATTTCGCTGCGTCCAAAG-3’	5’-GCTTCCGGAACAAACTGGGTAA-3’	135
Epcam	NM_008532	5’-CCACGTGCTGGTGTGTCAA-3’	5’-TCTGCAAGCTCTGATGGTCGTA-3’	150
c-Fos	NM_010234	5’-CAGAGCGGGAATGGTGAAGAC-3’	5’-CCGTTTCTCTTCCTCTTCAGGAGAT-3’	100
c-Jun	NM_010591	5’-AGAACTTGACTGGTTGCGACAGAG-3’	5’-AGGTCGCAACCCAGTCCATC-3’	84
c-Myc	NM_001177352	5’-TCGCCCAAATCCTGTACCTC-3’	5’-TCTCCACAGACACCACATCAAT-3’	193
Birc5	NM_009689	5’-AAGGAATTGGAAGGCTGGGAAC-3’	5’-TCTTCCATCTGCTTCTTGACAGTGA -3’	101
Ccnd1	NM_001379248	5’-CTGCATGTTCGTGGCCTCTA-3’	5’-GCCAGGTTCCACTTGAGCTTG-3’	144
Anxa2	NM_007585	5’-GGACATTGCCTTCGCCTATCA-3’	5’-CGTCTCCAGGTGGCCAGATAA-3’	85
Gapdh	NM_008084	5’-ATCAAGAAGGTGGTGAAGCA-3’	5’-CTGTAGCCGTATTCATTGTCA-3’	191

Supplemental Table S3. BA composition of total BA pool in wild type (WT) and *Cyp2a12/Cyp2c70* double knockout (DKO) mice

%	15 weeks				47 weeks			
	WT		DKO		WT		DKO	
	ND	HFHSD	ND	HFHSD	ND	HFHSD	ND	HFHSD
TCA	18.8 ± 1.5	27.6 ± 1.2[a]	5.5 ± 0.5[a]	14.1 ± 1.7[d,h]	13.8 ± 1.0	21.2 ± 2.6[c]	3.7 ± 1.1[a]	5.9 ± 1.5[d]
TωMCA	36.4 ± 0.6	25.5 ± 2.5[a]	0.0 ± 0.0[a]	0.0 ± 0.0[d]	35.2 ± 1.1	28.8 ± 1.4[a]	0.0 ± 0.0[a]	0.0 ± 0.0[d]
TαMCA	5.7 ± 0.4	10.9 ± 1.3[b]	0.0 ± 0.0[a]	0.0 ± 0.0[d]	5.1 ± 0.3	7.5 ± 0.6[a]	0.0 ± 0.0[a]	0.0 ± 0.0[d]
TβMCA	19.6 ± 1.2	22.5 ± 1.7	0.2 ± 0.0[a]	0.2 ± 0.0[d]	16.9 ± 0.6	22.1 ± 1.1[a]	0.7 ± 0.3[a]	0.1 ± 0.0[d]
TCDCA	0.7 ± 0.1	4.6 ± 0.9	33.2 ± 1.8[a]	38.1 ± 5.1[d]	1.1 ± 0.1	2.5 ± 0.3	35.3 ± 3.3[a]	58.0 ± 4.9[d,g]
TDCA	3.0 ± 0.3	1.7 ± 0.1	29.8 ± 2.2[a]	28.6 ± 5.0[d]	3.4 ± 0.3	3.2 ± 0.7	17.3 ± 3.6[a]	12.3 ± 3.4
TLCA	0.0 ± 0.0	0.0 ± 0.0	16.7 ± 1.0[a]	10.8 ± 1.2[d,g]	0.2 ± 0.0	0.0 ± 0.0	15.3 ± 1.3[a]	12.6 ± 1.4[d]
TUDCA	0.8 ± 0.1	1.7 ± 0.2	1.7 ± 0.2	1.8 ± 0.6	0.8 ± 0.0	2.0 ± 0.2	14.6 ± 4.4[a]	1.5 ± 0.4[h]
THDCA	0.8 ± 0.1	0.4 ± 0.0[a]	0.7 ± 0.1	0.4 ± 0.0[h]	1.0 ± 0.1	0.5 ± 0.1[b]	0.8 ± 0.1	1.0 ± 0.1[f]
GCA	0.2 ± 0.0	0.2 ± 0.0[c]	0.0 ± 0.0[a]	0.1 ± 0.0[e,h]	0.1 ± 0.0	0.1 ± 0.0	0.0 ± 0.0[b]	0.1 ± 0.0
GCDCA	0.0 ± 0.0	0.0 ± 0.0	0.1 ± 0.0[a]	0.1 ± 0.0[d]	0.0 ± 0.0	0.0 ± 0.0	0.1 ± 0.0[a]	0.1 ± 0.0[d]
GDCA	0.0 ± 0.0	0.0 ± 0.0	0.1 ± 0.0[a]	0.1 ± 0.0[e]	0.0 ± 0.0	0.0 ± 0.0	0.0 ± 0.0	0.0 ± 0.0
GLCA	0.0 ± 0.0	0.0 ± 0.0	0.0 ± 0.0	0.0 ± 0.0	0.0 ± 0.0	0.0 ± 0.0	0.0 ± 0.0	0.0 ± 0.0
GUDCA	0.0 ± 0.0	0.0 ± 0.0	0.0 ± 0.0	0.0 ± 0.0	0.0 ± 0.0	0.0 ± 0.0	0.0 ± 0.0	0.0 ± 0.0
CA	8.6 ± 1.7	3.1 ± 0.5[c]	2.1 ± 0.7[b]	2.1 ± 0.8	10.6 ± 0.9	6.9 ± 0.9[c]	1.6 ± 0.5[a]	2.2 ± 0.9[e]
ωMCA	1.1 ± 0.2	0.4 ± 0.1[b]	0.0 ± 0.0[a]	0.0 ± 0.0	2.8 ± 0.3	1.6 ± 0.3[b]	0.0 ± 0.0[a]	0.0 ± 0.0[e]
αMCA	0.4 ± 0.1	0.3 ± 0.0	0.0 ± 0.0[a]	0.0 ± 0.0[e]	0.9 ± 0.1	0.8 ± 0.1	0.0 ± 0.0[a]	0.0 ± 0.0[d]
βMCA	1.2 ± 0.2	0.8 ± 0.2	0.0 ± 0.0[a]	0.0 ± 0.0[f]	2.7 ± 0.3	2.5 ± 0.3	0.0 ± 0.0[a]	0.0 ± 0.0[d]
HCA	0.0 ± 0.0	0.0 ± 0.0	0.0 ± 0.0	0.0 ± 0.0	0.0 ± 0.0	0.0 ± 0.0	0.0 ± 0.0	0.0 ± 0.0
CDCA	0.1 ± 0.0	0.1 ± 0.0	3.8 ± 1.3[c]	2.0 ± 0.9	0.2 ± 0.0	0.2 ± 0.0	4.1 ± 1.4[b]	4.0 ± 1.1[f]
DCA	0.4 ± 0.1	0.1 ± 0.0	3.5 ± 1.4	0.8 ± 0.3	0.5 ± 0.1	0.2 ± 0.0	1.2 ± 0.3	0.7 ± 0.3
LCA	0.0 ± 0.0	0.0 ± 0.0	1.4 ± 0.4[b]	0.3 ± 0.1	0.0 ± 0.0	0.0 ± 0.0	1.5 ± 0.5[b]	0.4 ± 0.0
UDCA	0.1 ± 0.0	0.0 ± 0.0	0.1 ± 0.0	0.1 ± 0.0	0.1 ± 0.0	0.2 ± 0.0	2.2 ± 0.9[b]	0.2 ± 0.0[i]
MDCA	0.1 ± 0.0	0.0 ± 0.0	0.1 ± 0.0	0.0 ± 0.0	0.1 ± 0.0	0.1 ± 0.0	0.1 ± 0.0	0.1 ± 0.0
HDCA	0.1 ± 0.0	0.0 ± 0.0	0.1 ± 0.0	0.0 ± 0.0	0.1 ± 0.0	0.0 ± 0.0[c]	0.1 ± 0.0	0.1 ± 0.0
7oxo-LCA	0.0 ± 0.0	0.0 ± 0.0	0.0 ± 0.0	0.0 ± 0.0	0.0 ± 0.0	0.0 ± 0.0	0.0 ± 0.0	0.0 ± 0.0
12oxo-LCA	0.0 ± 0.0	0.0 ± 0.0	0.1 ± 0.0	0.0 ± 0.0	0.0 ± 0.0	0.0 ± 0.0	0.0 ± 0.0	0.0 ± 0.0[i]
12oxo-CDCA	0.3 ± 0.1	0.0 ± 0.0[b]	0.0 ± 0.0[b]	0.0 ± 0.0	0.7 ± 0.1	0.1 ± 0.0[a]	0.0 ± 0.0[a]	0.0 ± 0.0
7oxo-DCA	1.6 ± 0.4	0.0 ± 0.0[b]	0.3 ± 0.1[b]	0.0 ± 0.0	3.1 ± 0.4	0.1 ± 0.0[a]	0.3 ± 0.1[a]	0.0 ± 0.0
3deH-LCA	0.0 ± 0.0	0.0 ± 0.0	0.1 ± 0.0[b]	0.1 ± 0.0	0.0 ± 0.0	0.0 ± 0.0	0.2 ± 0.0[b]	0.1 ± 0.0
3deH-DCA	0.0 ± 0.0	0.0 ± 0.0	0.1 ± 0.0[c]	0.1 ± 0.0	0.0 ± 0.0	0.0 ± 0.0	0.1 ± 0.0	0.1 ± 0.0
3deH-CDCA	0.0 ± 0.0	0.0 ± 0.0	0.0 ± 0.0	0.1 ± 0.0	0.0 ± 0.0	0.0 ± 0.0	0.1 ± 0.0[c]	0.1 ± 0.0[f]
3deH-UDCA	0.0 ± 0.0	0.0 ± 0.0	0.0 ± 0.0	0.0 ± 0.0	0.0 ± 0.0	0.0 ± 0.0	0.0 ± 0.0	0.0 ± 0.0
3deH-CA	0.0 ± 0.0	0.0 ± 0.0	0.0 ± 0.0	0.0 ± 0.0	0.0 ± 0.0	0.0 ± 0.0	0.0 ± 0.0	0.0 ± 0.0
7epi-CA	0.2 ± 0.0	0.0 ± 0.0[c]	0.1 ± 0.0	0.0 ± 0.0	0.3 ± 0.1	0.0 ± 0.0[a]	0.0 ± 0.0[a]	0.0 ± 0.0
12epi-CA	0.0 ± 0.0	0.0 ± 0.0	0.0 ± 0.0	0.0 ± 0.0	0.0 ± 0.0	0.0 ± 0.0	0.4 ± 0.3	0.0 ± 0.0
12epi-DCA	0.0 ± 0.0	0.0 ± 0.0	0.1 ± 0.0	0.0 ± 0.0	0.2 ± 0.0	0.0 ± 0.0	0.0 ± 0.0	0.0 ± 0.0
3epi-CDCA+DCA	0.0 ± 0.0	0.0 ± 0.0	0.0 ± 0.0	0.0 ± 0.0	0.0 ± 0.0	0.0 ± 0.0	0.0 ± 0.0	0.0 ± 0.0
3epi-UDCA	0.0 ± 0.0	0.0 ± 0.0	0.0 ± 0.0	0.0 ± 0.0	0.0 ± 0.0	0.0 ± 0.0	0.0 ± 0.0	0.0 ± 0.0
3epi-LCA	0.0 ± 0.0	0.0 ± 0.0	0.0 ± 0.0	0.0 ± 0.0	0.0 ± 0.0	0.0 ± 0.0	0.1 ± 0.0[a]	0.1 ± 0.0[f]
Total	100	100	100	100	100	100	100	100

ND, normal chow diet; HFHSD, high-fat/high-sucrose diet.

Data are expressed as mean ± SEM.

[a]$p<0.001$, [b]$p<0.01$, and [c]$p<0.05$ versus WT (ND) by Tukey-Kramer test.

[d]$p<0.001$, [e]$p<0.01$, and [f]$p<0.05$ versus WT (HFHSD) by Tukey-Kramer test.

[g]$p<0.001$, [h]$p<0.01$, and [i]$p<0.05$ versus DKO (ND) by Tukey-Kramer test.

Supplemental Table S4. BA concentrations in the liver, gallbladder, and small intestine (µmol/whole organ) and their sum (total BA pool size) in wild type (WT) and *Cyp2a12/Cyp2c70* double knockout (DKO) mice

	15 weeks				47 weeks			
	WT		DKO		WT		DKO	
	ND	HFHSD	ND	HFHSD	ND	HFHSD	ND	HFHSD
Liver	0.1 ± 0.0	0.2 ± 0.0	0.2 ± 0.1	0.5 ± 0.2	0.1 ± 0.0	0.7 ± 0.1[b]	0.6 ± 0.1[b]	1.1 ± 0.1[e]
	(0.6 ± 0.1)	(1.5 ± 0.3)	(2.7 ± 0.6)	(5.2 ± 2.1[d])	(1.1 ± 0.1)	(5.4 ± 0.6[b])	(4.4 ± 1.1 [b])	(7.8 ± 0.7[f])
Gallbladder	1.1 ± 0.2	1.0 ± 0.2	2.8 ± 0.8	1.7 ± 0.9	0.8 ± 0.2	0.4 ± 0.1	4.4 ± 1.1 [b]	0.9 ± 0.5[e]
	(7.4 ± 1.4)	(7.0 ± 2.4)	(31.8 ± 7.8[c])	(15.2 ± 8.4)	(8.8 ± 1.8)	(3.3 ± 1.0)	(27.3 ± 5.6[b])	(5.5 ± 2.9[e])
Small intestine	13.7 ± 0.8	14.9 ± 1.8	5.8 ± 0.8[b]	8.5 ± 0.7[d]	8.0 ± 0.6	11.5 ± 1.2	10.0 ± 1.1	12.2 ± 0.7
	(92.0 ±1.3)	(91.5 ± 2.7)	(65.5 ± 7.7[b])	(79.6 ± 6.5)	(90.1 ± 1.8)	(91.3 ± 0.5)	(68.3 ± 6.0[a])	(86.7 ± 2.8[e])
Total pool	14.9 ± 0.7	16.1 ± 1.6	8.9 ± 0.5[b]	10.7 ± 0.6[d]	8.9 ± 0.6	12.6 ± 1.3	15.1 ± 1.9[b]	14.2 ± 1.0
	(100)	(100)	(100)	(100)	(100)	(100)	(100)	(100)

ND, normal chow diet; HFHSD, high-fat/high-sucrose diet.

Data are expressed as mean ± SEM. Proportion (%) is shown in parenthesis.

[a]$p<0.001$, [b]$p<0.01$, and [c]$p<0.05$ versus WT (ND) by Tukey-Kramer test.

[d]$p<0.05$ versus WT (HFHSD) by Tukey-Kramer test.

[e]$p<0.01$ and [f]$p<0.05$ versus DKO (ND) by Tukey-Kramer test.

Supplemental Table S5. Hepatic oxysterol concentrations in wild type (WT) and *Cyp2a12/Cyp2c70* double knockout (DKO) mice

ng/mg protein	15 weeks				47 weeks			
	WT		DKO		WT		DKO	
	ND	HFHSD	ND	HFHSD	ND	HFHSD	ND	HFHSD
22R-HC	0.1 ± 0.0	0.1 ± 0.0	0.1 ± 0.0	0.1 ± 0.0	0.1 ± 0.0	0.1 ± 0.0	0.2 ± 0.1	0.5 ± 0.2[b]
24S-HC	0.2 ± 0.0	0.4 ± 0.1[b]	0.2 ± 0.0	0.2 ± 0.0[e]	0.2 ± 0.0	1.0 ± 0.2[b]	0.2 ± 0.1	0.5 ± 0.2
24S,25-EC	0.0 ± 0.0	0.2 ± 0.0[c]	0.1 ± 0.0	0.2 ± 0.0[b]	0.1 ± 0.0	0.3 ± 0.1	0.2 ± 0.1	0.3 ± 0.1
25-HC	0.1 ± 0.0	0.2 ± 0.1[c]	0.1 ± 0.0	0.4 ± 0.1[a,e,f]	0.2 ± 0.0	0.9 ± 0.1[a]	0.2 ± 0.0	0.6 ± 0.1[b,g]
27-HC	2.2 ± 0.2	2.3 ± 0.4	2.1 ± 0.4	3.0 ± 0.3	3.4 ± 0.3	4.2 ± 0.3	2.5 ± 0.6	3.4 ± 1.2
4β-HC	5.5 ± 0.5	2.3 ± 0.3	14.8 ± 0.8[b]	11.5 ± 2.9[d]	6.7 ± 0.3	8.3 ± 1.0	31.2 ± 6.6[c]	46.8 ± 10.1[a,d]
7α-HC	0.5 ± 0.0	0.9 ± 0.1	0.6 ± 0.0	1.7 ± 0.4[b,e,g]	1.5 ± 0.2	4.1 ± 1.2	1.5 ± 0.4	6.9 ± 1.8[b,g]
7β-HC	0.3 ± 0.0	0.4 ± 0.0	0.4 ± 0.0	1.0 ± 0.2[b,e,i]	1.5 ± 0.2	2.3 ± 0.6	1.7 ± 0.7	4.9 ± 1.3[c,h]
7-OC	3.4 ± 0.4	12.7 ± 2.4[b]	2.0 ± 0.1	5.5 ± 1.4[f]	3.3 ± 0.2	13.7 ± 3.1[c]	4.2 ± 0.9	24.2 ± 5.8[a,f]
5α6α-EC	0.5 ± 0.1	1.0 ± 0.2	0.6 ± 0.1	1.2 ± 0.3	0.8 ± 0.1	2.3 ± 0.6	1.1 ± 0.2	4.4 ± 1.1[a,e,f]
5β6β-EC	1.2 ± 0.1	2.9 ± 0.4	1.4 ± 0.2	3.3 ± 0.9[c]	1.5 ± 0.2	6.8 ± 1.5	2.4 ± 0.4	12.2 ± 3.3[a,f]
3β5α6β-Triol	0.1 ± 0.0	0.1 ± 0.0	0.2 ± 0.0[c]	0.3 ± 0.0[a,d,h]	0.4 ± 0.0	1.30 ± 0.4	0.3 ± 0.10	2.4 ± 0.8[b,g]

ND, normal chow diet; HFHSD, high-fat/high-sucrose diet; HC, hydroxycholesterol; OC, oxocholesterol; EC, epoxycholesterol (or epoxycholestanol).

Oxysterols in green letters are ligands for LXR, while those in red are autoxidation products of cholesterol.

Data are expressed as mean ± SEM.

[a]p<0.001, [b]p<0.01, and [c]p<0.05 versus WT (ND) by Tukey-Kramer test.

[d]p<0.01 and [e]p<0.05 versus WT (HFHSD) by Tukey-Kramer test.

[f]p<0.001, [g]p<0.01, and [h]p<0.05 versus DKO (ND) by Tukey-Kramer test.

Supplemental Table S6. Serum concentrations of organic compounds in wild type (WT) and *Cyp2a12/Cyp2c70* double knockout (DKO) mice

μg/mL	15 weeks				47 weeks			
	WT		DKO		WT		DKO	
	ND	HFHSD	ND	HFHSD	ND	HFHSD	ND	HFHSD
3-HB	224 ± 26	148 ± 14	192 ± 31	104 ± 11^{c}	161 ± 15	54 ± 14^{a}	73 ± 12^{a}	34 ± 3^{a}
3-HIB	11.6 ± 0.5	5.8 ± 0.3^{c}	17.1 ± 1.2^{c}	6.4 ± 1.8^{c,d}	14.7 ± 0.8	4.3 ± 0.6^{b}	15.4 ± 3.0	4.3 ± 0.8^{b,e}
Carnitine	3.2 ± 0.3	3.5 ± 0.1	2.6 ± 0.2	3.9 ± 0.5	3.4 ± 0.3	6.9 ± 0.4	5.9 ± 1.0	10.4 ± 3.1^{b}
Acetylcarnitine	8.2 ± 1.0	5.5 ± 0.3	5.9 ± 0.7	4.6 ± 0.7^{c}	8.1 ± 0.4	4.1 ± 0.5^{b}	6.3 ± 0.9	4.9 ± 1.3^{c}
Choline	5.8 ± 0.3	6.5 ± 0.6	6.1 ± 0.3	6.2 ± 0.7	5.9 ± 0.4	13.3 ± 1.8^{c}	8.8 ± 1.5	16.0 ± 2.9^{b,f}
TMAO	0.22 ± 0.02	0.16 ± 0.01	0.27 ± 0.03	0.17 ± 0.03^{f}	0.36 ± 0.04	0.11 ± 0.02	0.65 ± 0.13	0.24 ± 0.04^{f}
Taurine	89 ± 6	74 ± 6	102 ± 4	85 ± 5	102 ± 7	120 ± 9	120 ± 12	134 ± 27

ND, normal chow diet; HFHSD, high-fat/high-sucrose diet; 3-HB, 3-hydroxybutyrate; 3-HIB, 3-hydroxyisobutyrate; TMAO, trimethylamine N-oxide.

Data are expressed as mean ± SEM.

$^{a}p<0.001$, $^{b}p<0.01$, and $^{c}p<0.05$ versus WT (ND) by Tukey-Kramer test.

$^{d}p<0.001$, $^{e}p<0.01$, and $^{f}p<0.05$ versus DKO (ND) by Tukey-Kramer test.

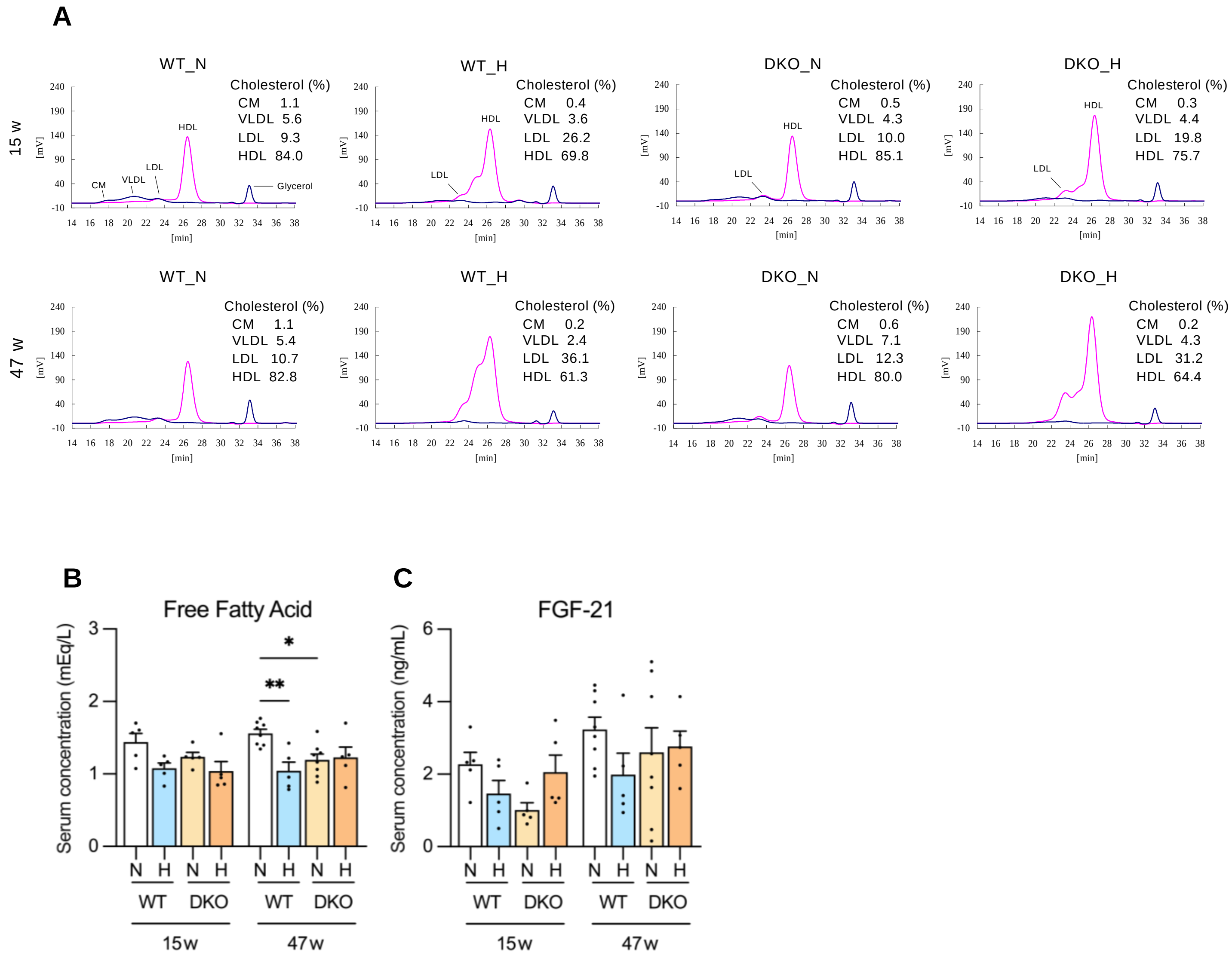

SUPPLEMENTAL FIGURE S1 Serum lipid analysis and FGF-21 concentrations in male wild type (WT) and *Cyp2a12/Cyp2c70* double knockout (DKO) mice treated with different diets. (A) Serum lipoprotein profiles determined by gel permeation HPLC. (B) Serum free fatty acid and (C) FGF-21 concentrations. Data are expressed as mean and SEM. *P < 0.05 and **P < 0.01 were considered significantly different by one-way ANOVA with post hoc Tukey-Kramer test. Abbreviations: N, normal diet; H, high-fat/high-sucrose diet (HFHSD); CM, chylomicron.

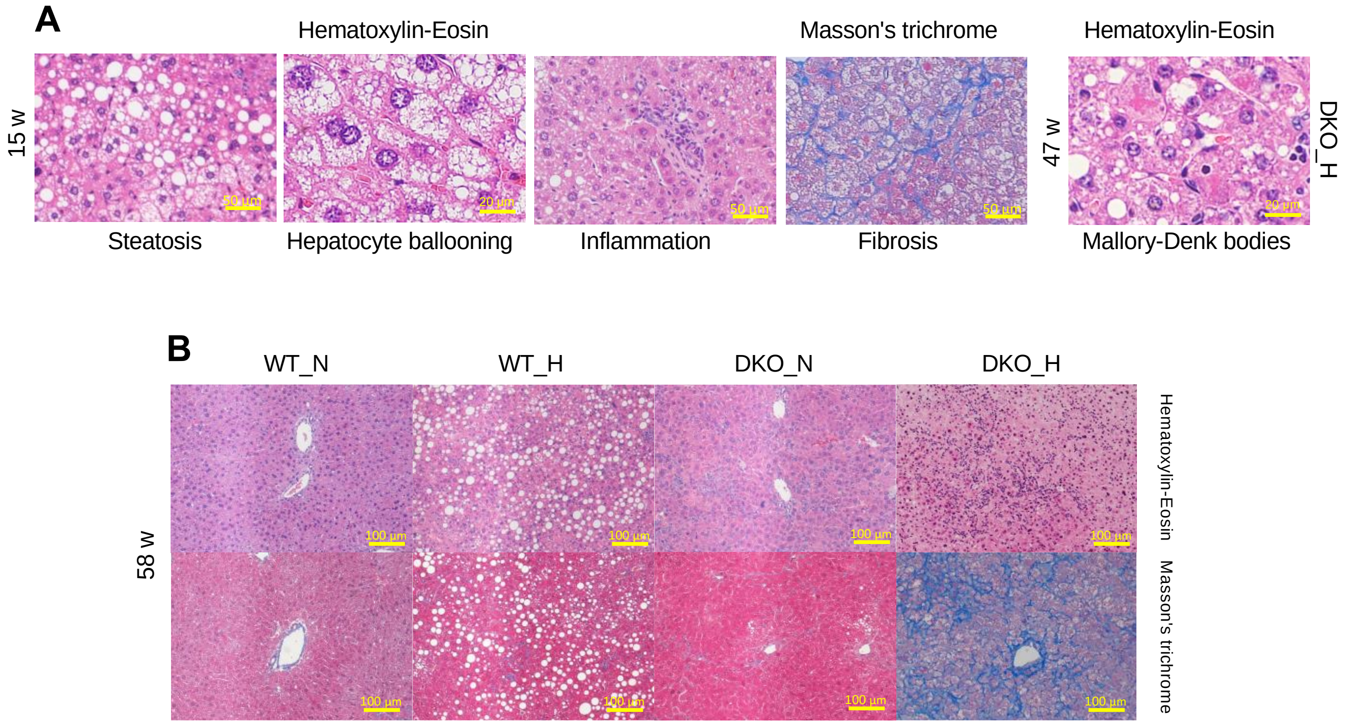

SUPPLEMENTAL FIGURE S2 Microscopic images of the livers from male wild type (WT) and *Cyp2a12/Cyp2c70* double knockout (DKO) mice treated with different diets. (A) Representative images of the livers from DKO mice treated with high-fat/high-sucrose diet (HFHSD) for 15 and 47 weeks. (B) Microscopic views of the livers from WT and DKO mice treated with normal diet (ND) or HFHSD for 58 weeks. Abbreviations: N, normal diet (ND); H, high-fat/high-sucrose diet (HFHSD).

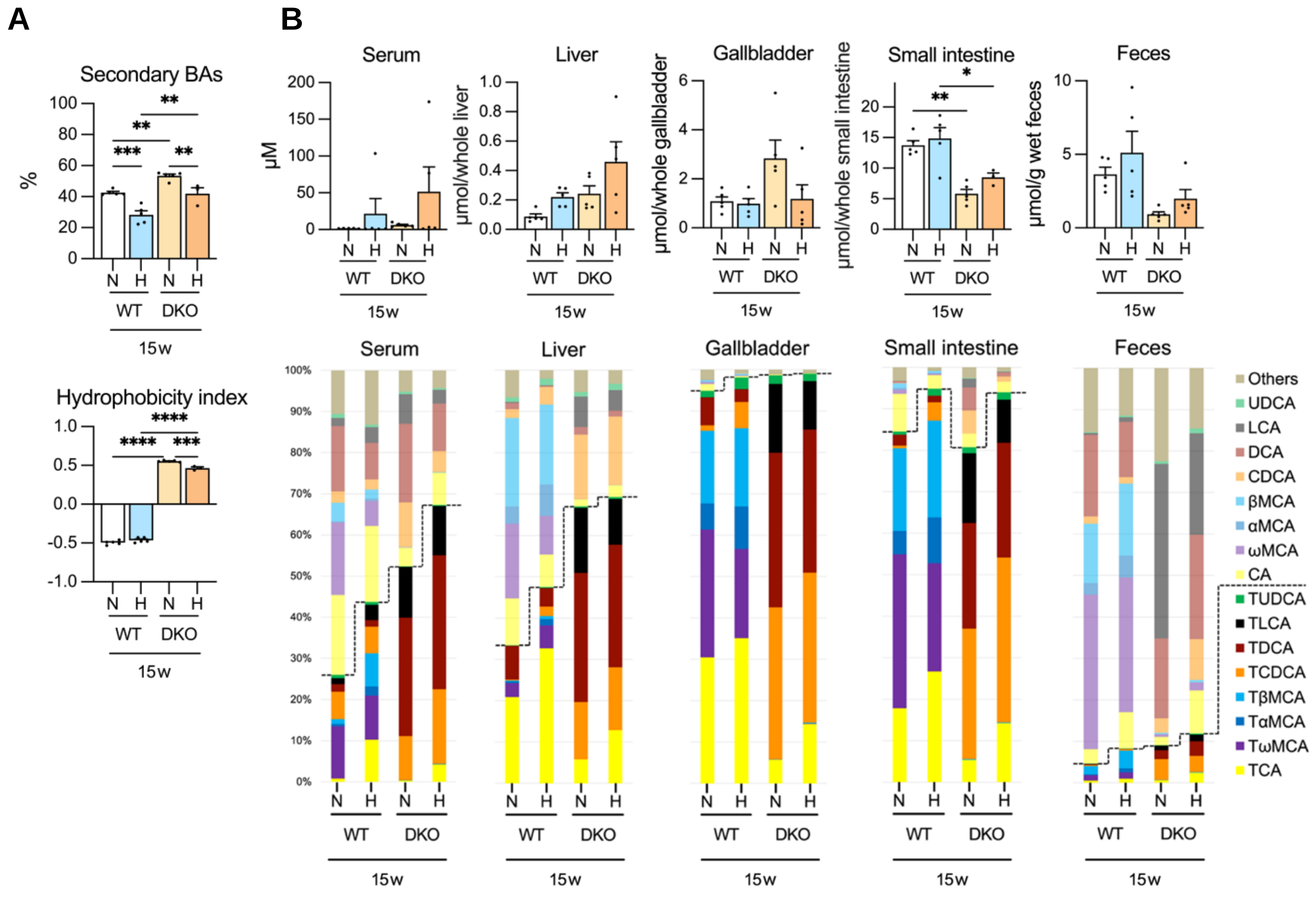

SUPPLEMENTAL FIGURE S3 Bile acid (BA) pool dynamics and composition in male wild-type (WT) and *Cyp2a12/Cyp2c70* double knockout (DKO) mice fed the indicated diets for 15 weeks. (A) Secondary BA percentages and hydrophobicity indices. (B) Comparison of serum, liver, gallbladder, small intestine, and feces BA composition. Data are presented as mean ± SEM. *, **, ***, and **** indicate $P < 0.05$, < 0.01, < 0.001, and < 0.0001, respectively, based on one-way ANOVA with a post hoc Tukey–Kramer test. N: normal diet, H: high-fat/high-sucrose diet.

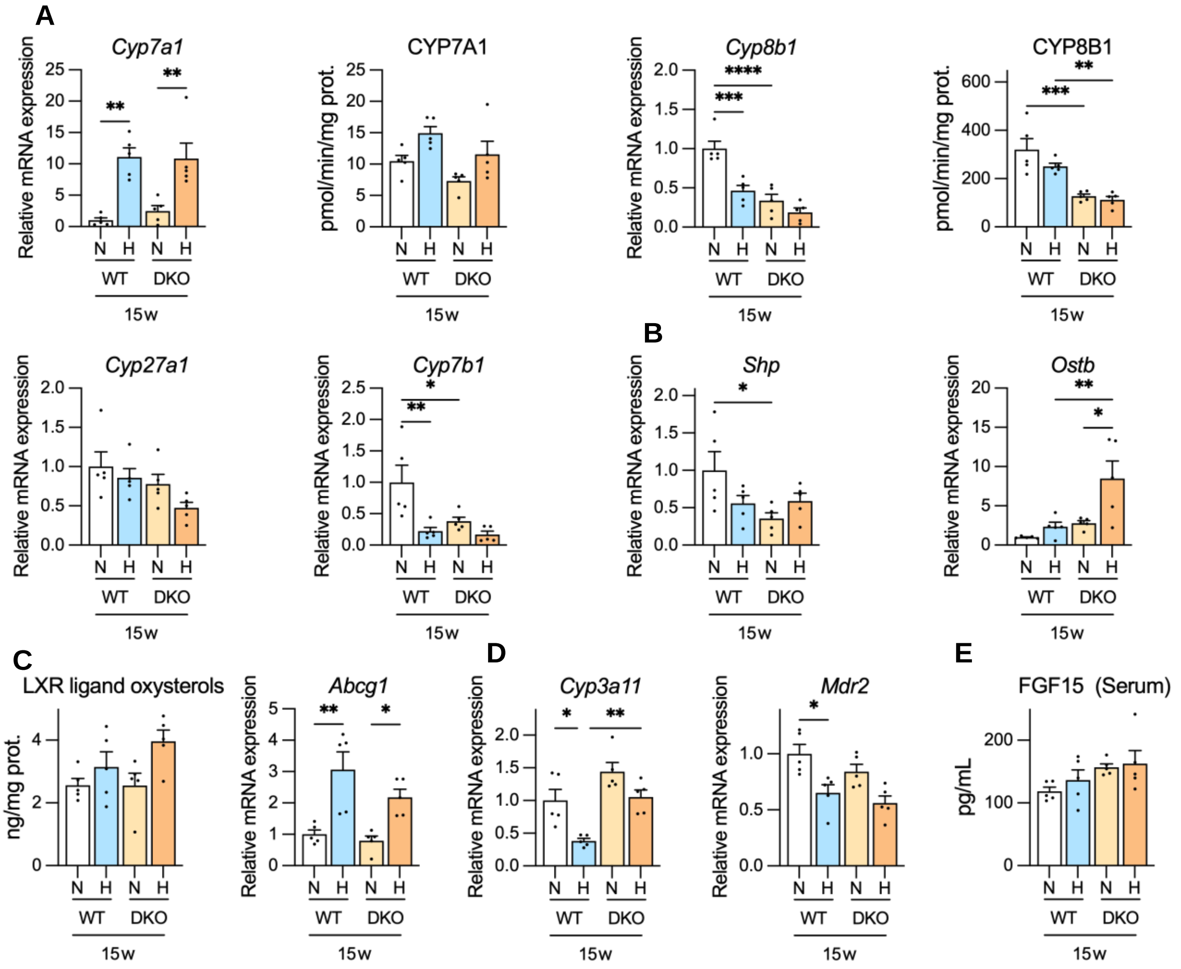

SUPPLEMENTAL FIGURE S4 Liver cholesterol and bile acid (BA) metabolism regulation in male wild-type (WT) and *Cyp2a12/Cyp2c70* double knockout (DKO) mice fed the indicated diets for 15 weeks. (A) Liver mRNA expression levels and activities of key enzymes in the BA biosynthesis pathway. (B) Liver mRNA levels of farnesoid X receptor (FXR) target genes. (C) Hepatic liver X receptor (LXR) ligand oxysterol levels and the mRNA levels of the LXR target gene, *Abcg1*. (D) Liver mRNA levels of the pregnane X receptor (PXR) target gene, *Cyp3a11*, and the peroxisome proliferator-activated receptor α (PPARα) target gene, *Mdr2*. (E) Serum FGF15 concentrations. Data are presented as mean ± SEM. *, **, ***, and **** indicate $P < 0.05$, < 0.01, < 0.001, and < 0.0001, respectively, based on one-way ANOVA with a post hoc Tukey–Kramer test. N: normal diet, H: high-fat/high-sucrose diet, Shp: small heterodimer partner, Ostb: organic solute transporter β, Abcg1: ATP-binding cassette transporter G1, Mdr2: multidrug resistance protein 2.

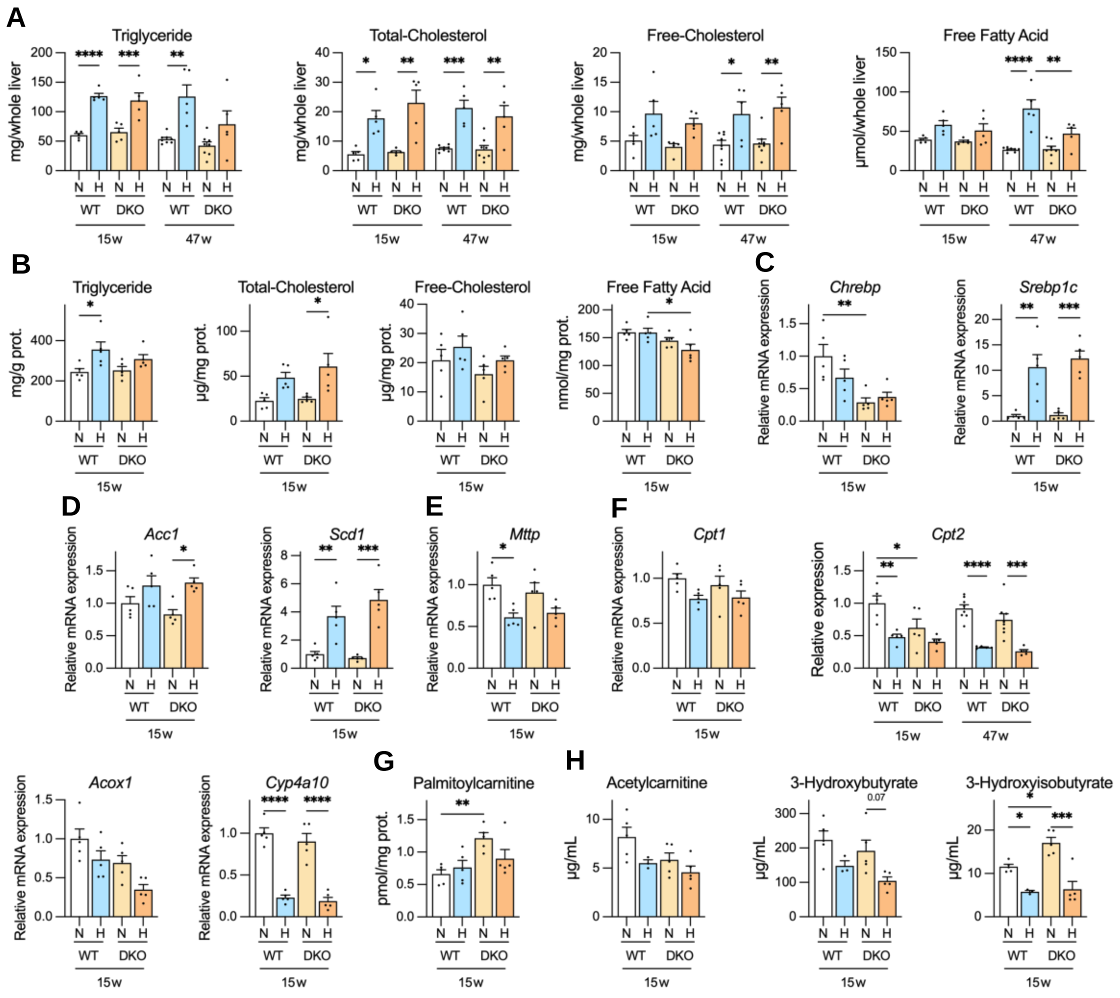

SUPPLEMENTAL FIGURE S5 Liver sugar, lipid, and amino acid metabolism regulation in male wild-type (WT) and *Cyp2a12/Cyp2c70* double knockout (DKO) mice fed the indicated diets for 15 or 47 weeks. (A) Liver lipid concentrations expressed per whole liver. (B) Liver lipid concentrations are expressed as per mg of protein. (C) Liver mRNA levels of fatty acid biosynthesis transcription factors, (D) key enzymes in *de novo* fatty acid synthesis, (E) microsomal triglyceride transfer protein (Mttp), which is crucial for VLDL synthesis, and (F) essential enzymes in fatty acid β- and ω-oxidation. (G) Liver levels of palmitoylcarnitine, a key intermediate in mitochondrial β-oxidation. (H) The serum levels of markers involved in fatty acid β-oxidation and amino acid degradation. Data are presented as mean ± SEM. *, **, ***, and **** indicate $P < 0.05$, < 0.01, < 0.001, and < 0.0001, respectively, based on one-way ANOVA with a post hoc Tukey–Kramer test. N: normal diet, H: high-fat/high-sucrose diet, Chrebp: carbohydrate-responsive element-binding protein, Srebp1c: sterol regulatory element-binding protein 1c, Acc1: acetyl-CoA carboxylase 1, Scd1: stearoyl-CoA desaturase 1, Cpt1: carnitine palmitoyltransferase 1, Cpt2: carnitine palmitoyltransferase 2, Acox1: acyl-CoA oxidase 1.

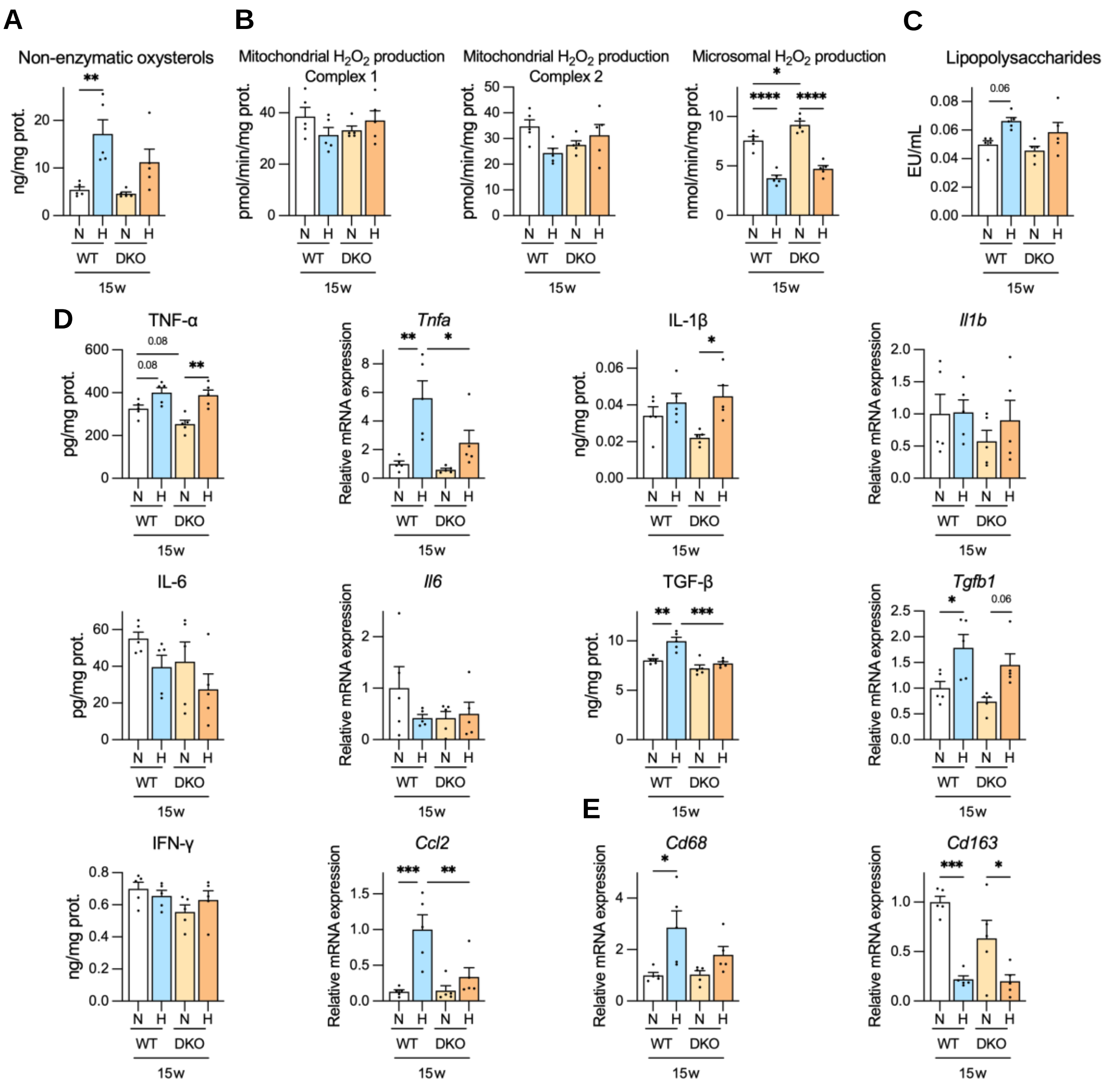

SUPPLEMENTAL FIGURE S6 Liver oxidative stress, ER stress, and inflammation regulation in male wild-type (WT) and *Cyp2a12/Cyp2c70* double knockout (DKO) mice fed the indicated diets for 15 weeks. (A) Liver levels of non-enzymatic oxysterols produced by oxidative stress. (B) Mitochondrial and microsomal H_2O_2 production. Pyruvate + malate (Complex I) or succinate in the presence of rotenone (Complex II) was used as mitochondrial oxidative substrates, and NADPH, in the presence of superoxide dismutase (SOD), was used as a microsomal oxidative substrate. (C) Serum lipopolysaccharide (LPS) concentrations. (D) Liver inflammatory cytokine concentrations and their mRNA levels. (E) Liver pan-macrophage marker, CD68, and M2 macrophage marker, CD163, mRNA levels. Data are presented as mean ± SEM. *, **, ***, and **** indicate $P < 0.05$, < 0.01, < 0.001, and < 0.0001, respectively, based on one-way ANOVA with a post hoc Tukey–Kramer test or Kruskal–Wallis with a post hoc Dunn–Bonferroni test. N: normal diet, H: high-fat/high-sucrose diet, ER: endoplasmic reticulum, IFN-γ: interferon γ, Ccl2: chemokine (C-C motif) ligand 2, Cd: cluster of differentiation.

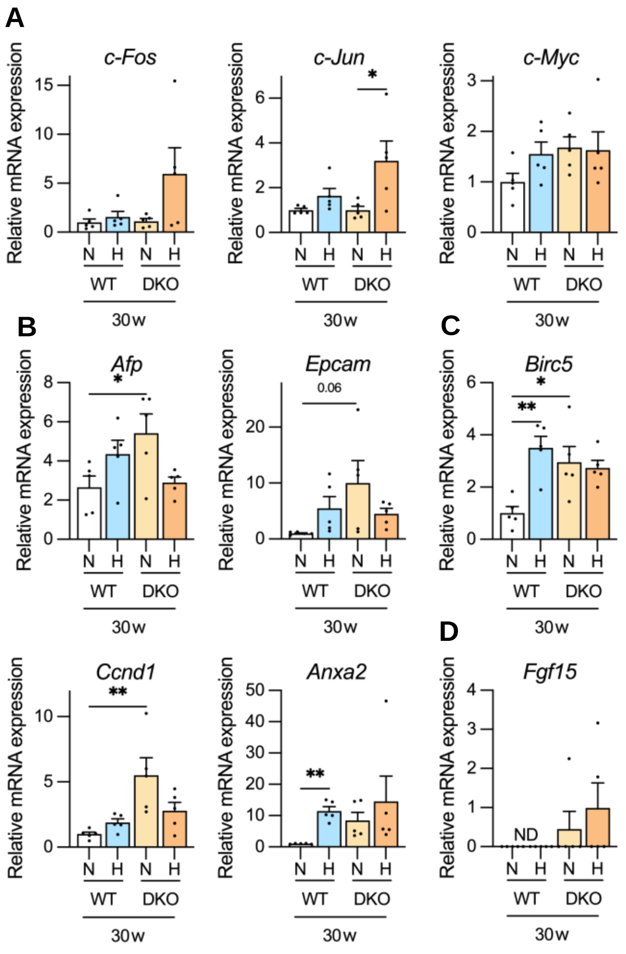

SUPPLEMENTAL FIGURE S7 Liver HCC-related oncogene regulation in male wild-type (WT) and *Cyp2a12/Cyp2c70* double knockout (DKO) mice fed the indicated diets for 30 weeks. Liver mRNA levels of (A) immediate early genes, (B) tumor markers, (C) STAT3 target genes, and (D) Fgf15. Data are presented as mean ± SEM. * and ** indicate $P < 0.05$ and < 0.01, respectively, based on one-way ANOVA with a post hoc Tukey–Kramer test or Kruskal–Wallis with a post hoc Dunn–Bonferroni test. N: normal diet, H: high-fat/high-sucrose diet, ND: not detected, Epcam: epithelial cell adhesion molecule, Birc5: baculoviral IAP repeat containing 5, Ccnd1: cyclin D1.

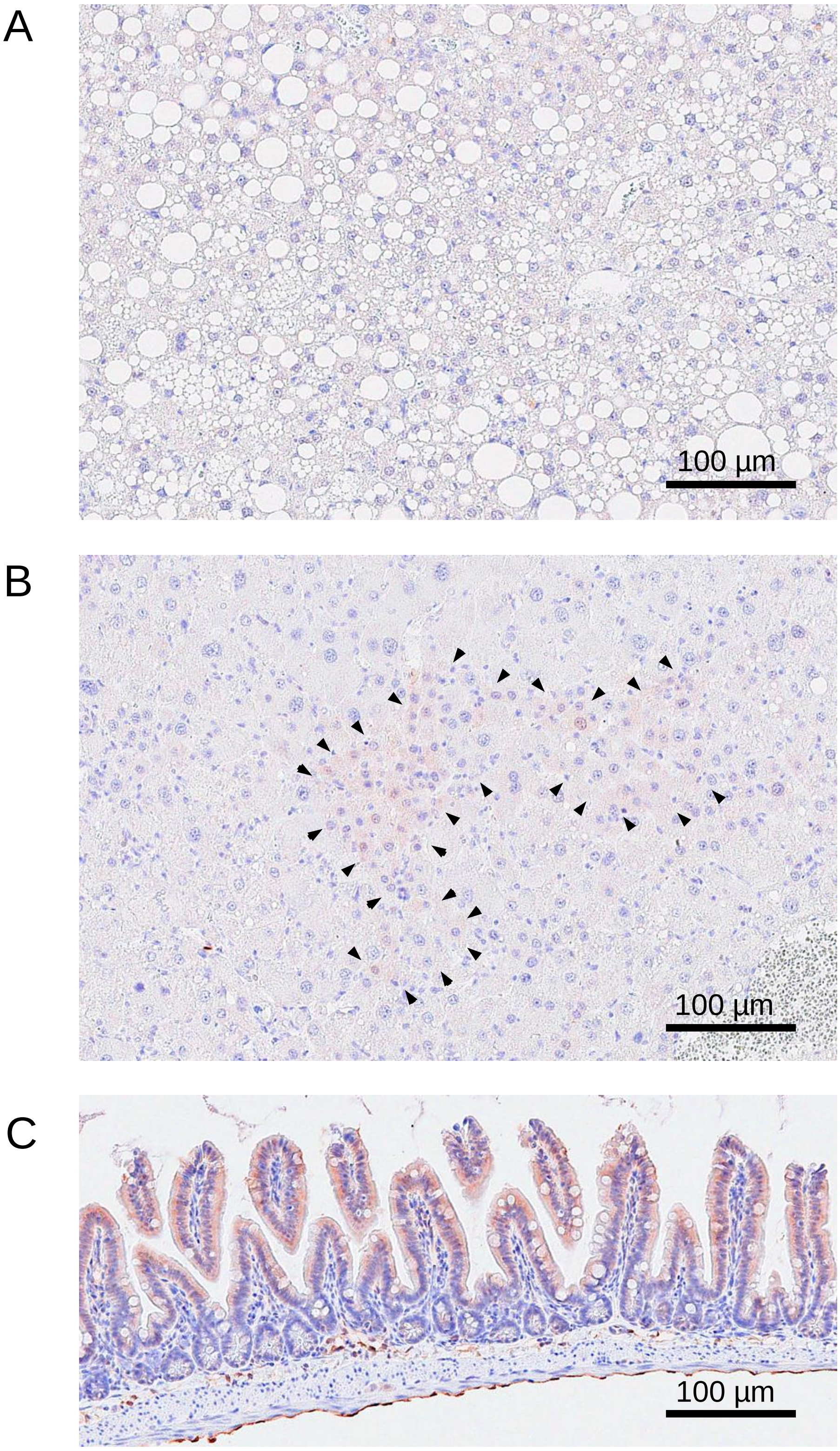

SUPPLEMENTAL FIGURE S8 Representative immunohistochemical staining for FGF15. (A) Liver from wild type (WT) mice treated with high-fat/high-sucrose diet (HFHSD) for 47 weeks. (B) Tumor area in the liver from Cyp2a12/Cyp2c70 double knockout (DKO) mice treated with HFHSD for 58 weeks. Arrowheads indicate a cluster of FGF15-high-expressing cells. (C) Ileum from WT mice for control staining.
